# Somehow I always end up alone: COVID-19, social isolation and crime in Queensland, Australia

**DOI:** 10.1186/s40163-020-00135-4

**Published:** 2020-11-24

**Authors:** Martin A. Andresen, Tarah Hodgkinson

**Affiliations:** 1 School of Criminology and Criminal Justice, Griffith University, Gold Coast Campus, Parklands Dr, Southport, QLD 4215 Australia; 2 School of Criminology and Criminal Justice, Griffith University, Mt Gravatt Campus, 176 Messines Ridge Road, Mt Gravatt, QLD 4122 Australia

**Keywords:** COVID-19, Crime, Queensland, Australia, Regional, Exceptional events

## Abstract

The COVID-19 pandemic has dramatically affected social life. In efforts to reduce the spread of the virus, countries around the world implemented social restrictions, including social distancing, working from home, and the shuttering of numerous businesses. These social restrictions have also affected crime rates. In this study, we investigate the impact of the COVID-19 pandemic on the frequency of offending (crimes include property, violent, mischief, and miscellaneous) in Queensland, Australia. In particular, we examine this impact across numerous settings, including rural, regional and urban. We measure these shifts across the restriction period, as well as the staged relaxation of these restrictions. In order to measure impact of this period we use structural break tests. In general, we find that criminal offences have significantly decreased during the initial lockdown, but as expected, increased once social restrictions were relaxed. These findings were consistent across Queensland’s districts, save for two areas. We discuss how these findings are important for criminal justice and social service practitioners when operating within an extraordinary event.

## Introduction

With its origins in Wuhan, China in late 2019, the COVID-19 pandemic has spread around the world (Readfern 2020). By the end of the first quarter of 2020, most nations had implemented social restrictions (social distancing, closing non-essential business, restricting local movement, etc.) in efforts to minimise the spread of the virus. Social interactions moved online, as did the economy, with the ways in which we interact changing radically, in many cases literally overnight. Aside from essential workers (health care providers, front-line officers, food services, etc.) and limited trips for groceries, medical concerns, and exercise, governments instructed residents to stay home.

Exceptional events, such as the COVID-19 global pandemic, though catastrophic in a number of dimensions, provide opportunities for natural experiments. These natural experiments can then be used to test our theories of human behaviour that can then be used to (hopefully) improve societal responses to future exceptional events, planned or otherwise. COVID-19 is considered an exceptional event because it impacts social structures and collective behaviour (Barton 1969). The introduction of social restrictions related to COVID-19 radically impacted human movement patterns (Google 2020) with significant reductions in movement away from the home.

Alongside these shifts in movement, many criminologists would expect changes in the opportunities for criminal activity. With more people spending the majority of their time at home, home guardianship would arguably improve and opportunities for crimes such as residential burglary would decrease. However, prolonged time spent at home by all residents, compounded by the financial stress of a global pandemic, could create additional opportunities for other crime types, such as domestic violence, as victim’s exposure to their offender increases (United Nations 2020).

Of additional interest is how social life and opportunities for crime may be impacted differently in different contexts. While much of the preliminary research on COVID-19 and crime has supported opportunity theories (Stickle and Felson 2020), few studies have examined the impact of COVID-19 in non-urban settings. For example, in rural settings, opportunities for crime types such as commercial burglary would be much lower, while opportunities for crime types such as agricultural equipment or stock theft might be much higher (Harkness 2017). Alternatively, in tourist destinations, there may be less guardianship against burglary as these properties may sit empty if their owners or renters are unable to travel (Mawby 2014). It is important then to examine the shifts in offending across different geographical contexts as a result of the COVID-19 pandemic to determine if certain areas may be at a higher risk of specific types of victimization.

In this paper, we investigate the impact COVID-19 on the opportunity structures for crime in Queensland, Australia. We identify changes that occur at the time of lockdown (social restrictions) as well as when the staged relaxation of those social restrictions occurred. We contribute to the research on COVID-19 and crime through the inclusion of this staged relaxation of social restrictions period as well as through an analysis of state-wide data in Queensland, Australia for 2 years. By analysing offence data across the entire state of Queensland, Australia separated into the 15 police districts, we are able to identify differential effects of social restrictions between urban, rural, and remote areas in Queensland. The implications for this research are to better understand the impacts of changing opportunity structures from imposed social restrictions that may improve planning for public safety with regard to future outbreaks of COVID-19 (currently underway in a number of countries) and future exceptional events.

## Related research

The impact of exceptional events on crime are understood through three theoretical frameworks: social cohesion/altruism, social disorganisation, and opportunity theories. Social cohesion/altruism approaches claim that during an exceptional event crime rates remain the same or decline, because people come together to help each other during a crisis (Barton 1969; Quarantelli 2007; Zahran et al. 2009). Empirical research has supported this in the context of both violent and property crime (Lemieux 2014; Siegel et al. 1999; Sweet 1998). It is important to note, however, that exceptional events often exacerbate social inequalities (Craemer 2010; Fothergill and Peek 2004). As such, others have argued that during an exceptional event social cohesion and collective efficacy are weakened by the social disorganization of crisis, impacting the ability of residents to control antisocial behaviour (Harper and Frailing 2012; Prelog 2016). Alternatively, to both of these proposals, opportunity-based explanations, such as the routine activity approach state that changes in crime are dependent on changes in the opportunity structure for crime (Hodgkinson and Andresen 2020).

Theoretical expectations vary from offence type to offence type and place to place: crime may increase because of decreased guardianship over businesses during a lockdown (commercial burglary), or crime may decrease because of the loss of opportunities under the same conditions (shoplifting) (Hodgkinson and Andresen 2020). In the case of COVID-19, the nature of the exceptional event is quite different. Unlike a typical natural disaster, that can create opportunities for disorganization and crime, as well as opportunities for altruism and social cohesion (Lemieux 2014) the COVID-19 pandemic has seen government systems actively discourage direct social interaction, but supporting other forms of social interaction in order to remain connected. This has created a particularly unique exceptional event type, which requires further investigation.

### COVID-19 and crime

Stickle and Felson (2020) and Eisner and Nivette (2020) have outlined a prospective research agenda that criminologists may undertake to understand the effects of COVID-19 and its social restrictions on criminal activity. In addition to this call for criminological research on a global-level natural experiment, reports and research are emerging discussing the impact of COVID-19 on crime—see Stickle and Felson (2020) for a discussion of the various research briefs that have emerged recently. Though preliminary in all cases due to the recency of changes in opportunity structures and that the effects of COVID-19 are far from over, this research has shown a number of interesting patterns that are consistent with expectations derived from changing opportunity structures (Stickle and Felson 2020).

Considering residential burglary in Detroit, Michigan, Felson et al. (2020) found that crime decreased significantly during the early stages of social restrictions. Perhaps most interesting, with implications for crime prevention, is that these decreases were greatest in areas with land use that are dominantly (> 90 percent) residential. In fact, areas with more mixed land use already saw increases in residential burglary by the end of March 2020. Mohler et al. (2020), in a study of a number of crime types in Los Angeles, California and Indianapolis, Indiana found that the changes in the volume of crime are not large in many cases. They did, however, find notable changes in robbery and traffic stops, with increases in domestic violence. Though instructive, these articles only considered data during the year 2020. As such, and (lack of) changes may be due to expected seasonal changes in their respective cities.

Borrion et al. (2020) investigated the impact of COVID-19 on retail theft in a city in China. Using a resilience framework, they found that retail theft decreased by over 60 percent, rebounding to a level higher than expected after social restrictions were relaxed. In an analysis of a variety of crime types across 16 cities in the United States, Ashby (2020) found no changes in serious assaults, decreases in residential burglary in some cities, decreases in theft from vehicles, inconsistent changes across cities for theft of vehicle, and little change in non-residential burglary. Of particular note is that Ashby (2020) found that results differed across the 16 cities under analysis: crime did not decrease in all cities and even increased in some cities. Hodgkinson and Andresen (2020) investigated a number of property, violence, and social disorder crimes in Vancouver, Canada. They found that most crime types decreased (or no increases based on expected seasonal patterns). Perhaps most interesting was a sudden drop in other theft (e.g. shoplifting) and a sharp increase and subsequent decrease in commercial burglary. Halford et al. (2020) used Google Covid-19 Community Mobility Reports to estimate the elasticity of crime to measure responsiveness to social restrictions imposed in a United Kingdom police service. They found that elasticities varied by crime type. In an analysis of domestic violence in Dallas, TX, Piquero et al. (2020a) found an initial spike, and subsequent decline, in the early stages of lockdown—see Reingle Gonzalez et al. (2020) and Piquero et al. (2020b) for discussions of these results. de la Miyar et al. (2020) investigated both conventional crime (domestic violence, burglary, and vehicle theft) as well as organized crime in Mexico City, finding that conventional crimes decreased but organized crime remained stable. And most closely related to the current research, Payne et al. (2020) analyzed violent crime for the state of Queensland, Australia, finding that violence dropped in the early stages of lockdown.

Though instructive, there are still many avenues of research to be undertaken. First, most of the existing research is based in North America—Stickle and Felson (2020) and Payne et al. (2020) do cite some research briefs that are outside of North America. And second, although there are a number of different cities, the areas studied are all urban areas or an entire state. However, if an opportunity approach is best suited to understanding crime trends in a pandemic, different opportunity structures need to be considered. In the analyses below, we consider all of the police districts in Queensland, Australia that include urban, regional and rural areas.

## Data and methods

**Fig. 1:**
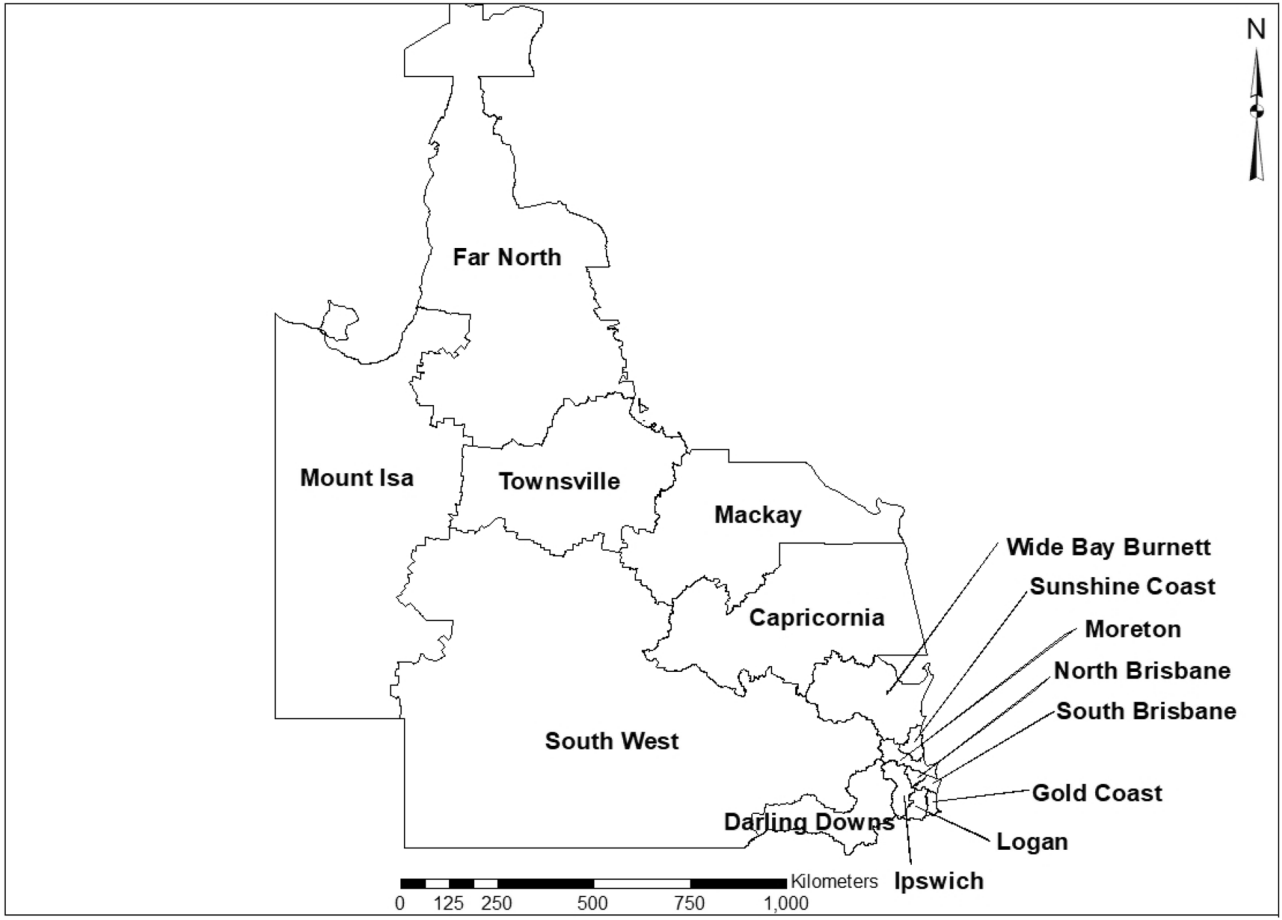
Queensland Police Service, Districts

We analyse social disorder, property, violent, and other offences across the entire state of Queensland, Australia. Queensland is approximately 1.85 million km^2^ (about 14 times larger than England) with a population of approximately 5 million people (less than one tenth the population of England), resulting in a fairly low population density outside of urban areas like Brisbane and the Gold Coast. The Queensland Police Service (QPS) organises the state into 15 districts of varying sizes, see Fig. 1. The majority of the Queensland population is in three of those districts: Gold Coast, North Brisbane, and South Brisbane, the predominantly urban areas. The remaining areas of Queensland, though containing small urban centres are mostly rural, regional and remote areas (Far North, Mount Isa, South West). Some areas on the eastern coast also contain popular vacation areas (Sunshine Coast) and tourist destinations such as Cairns in the Far North district.

**Fig. 2:**
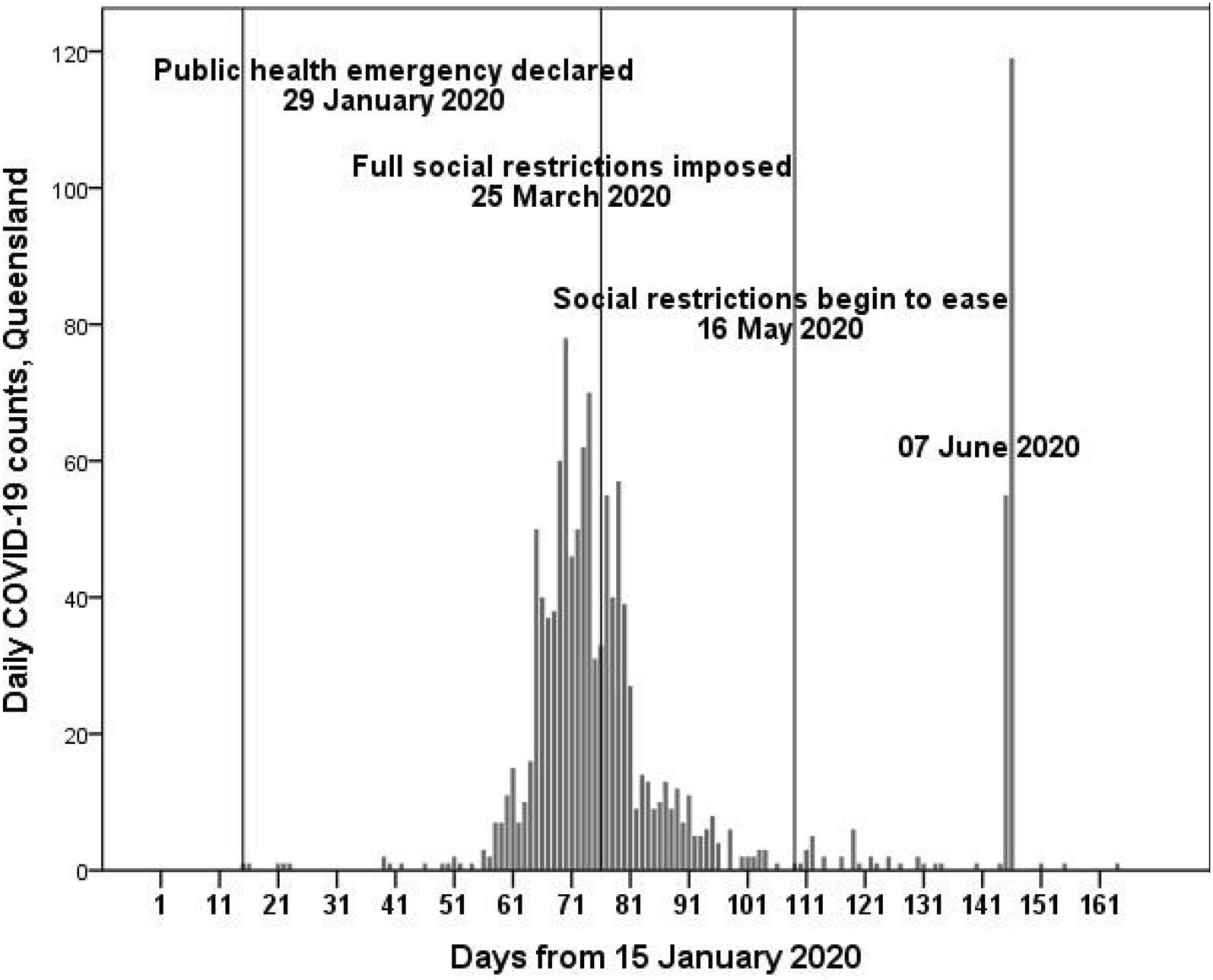
Daily COVID-19 infections, Queensland, 15 January 2020 to 02 July 2020

As shown in Fig. 2, Queensland has been successful with early social restrictions, in reducing COVID-19—the spike in June related to one person who worked on a large farm (ABC News 2020). Restrictions began with the declaration of a public health emergency the day after the first COVID-19 infection was identified in Queensland, 28 January 2020. On 19 March 2020 Australia banned arrivals of non-citizens and residents, with all non-essential services being shuttered 23 March 2020, and state borders being closed to all non-essential travel 25 March 2020. Stage 1 re-opening the economy began 02 May 2020, including the re-opening of restaurants, pubs and bars (up to 10 people) 2 weeks later. Stage 2 of the re-opening (up to 20 people in businesses and homes and full travel within Queensland) began 01 June 2020. And Stage 3 (largely normal business operations with COVID-19 safe planning) began 03 July 2020. Aside from the spike in early June 2020, the social restrictions imposed were very successful in reducing new COVID-19 infections: most days after 01 May 2020 had zero new infections.

**Fig. 3:**
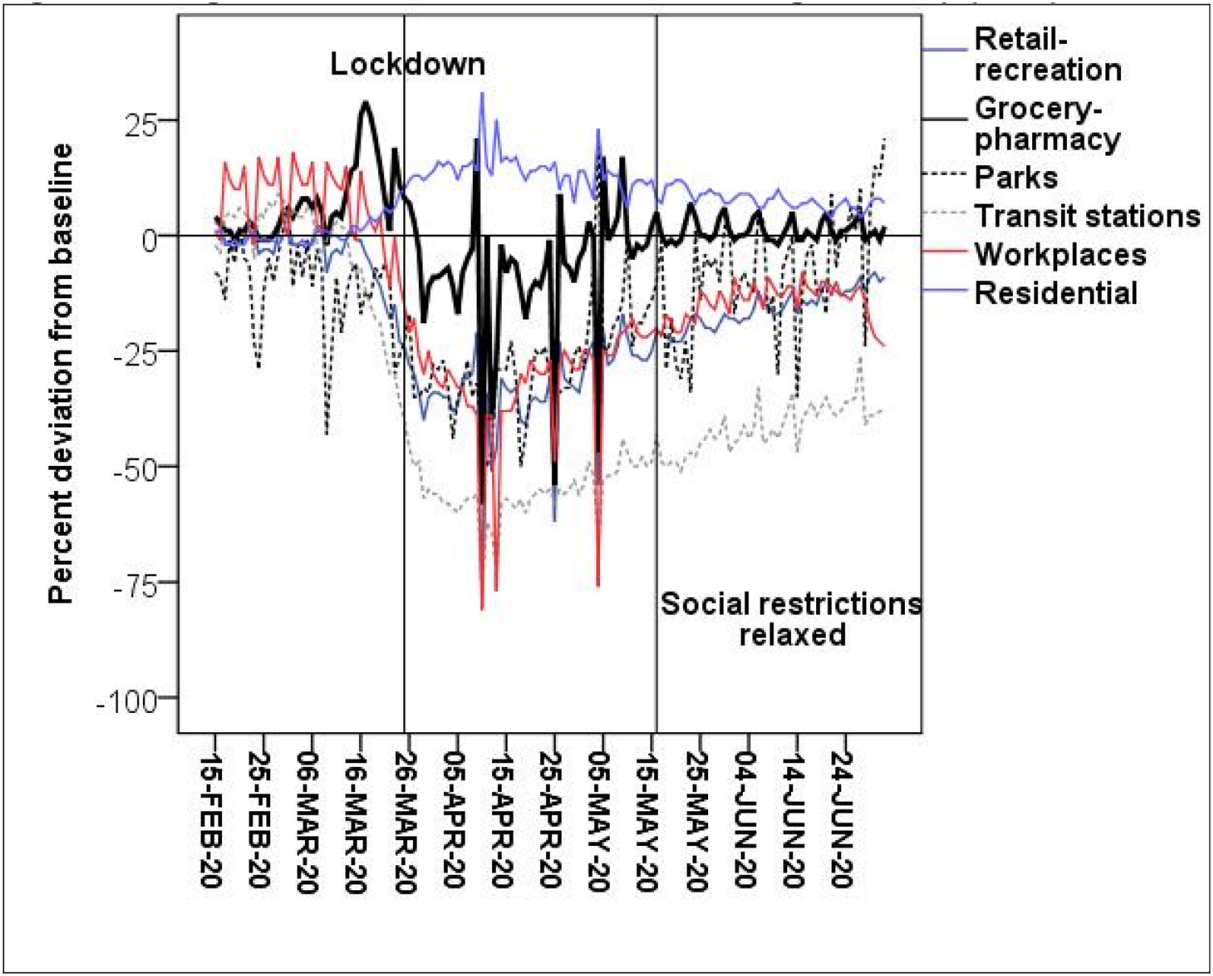
Changes in routine activities, Queensland, Google Mobility (2020) data

We expect that offences will decrease during the lockdown period, and begin to increase again during the staged relaxation of social restrictions. These reductions and following increases are based on changes in the opportunities for crime. This is evident in Fig. 3, that shows time spent in Queensland by location (Google 2020). This figure clearly shows that all activities outside of “residential” decreased before or immediately after lockdown. And during the relaxation of social restrictions, many of these activities began to return to their baseline level, with grocery-pharmacy returning to its baseline at this time. However, there may be some offences that may not change in this pattern. For example, because of the Queensland border restrictions, the QPS districts that border other Australian states may experience increases in traffic-related offences during both lockdown and staged relaxation of social restrictions because the borders remained closed at this time. This may be particularly the case because the Queensland Police Service modified its service delivery with up to 10 percent of its officers being deployed specifically to COVID-19 related duties (Crockford and Lynch 2020).

### Data

We use open source data provided from QPS: https://www.police.qld.gov.au/maps-and-statistics. Our data are measured weekly from 03 May 2018 through to 02 July 2020, 113 observations; 03 May 2018 is the first available data for QPS districts at the time of data collection and 02 July 2020 is the latest available data at the time of data collection but also when social restrictions entered another stage (becoming less restrictive). Of the 19 available offence types, we investigate any changes in the following: good order (social disorder),^1^ mischief (property damage), assault, robbery, other violence,^2^ total violence (the sum of assault, robbery, and other violence), burglary, theft, theft of vehicle (TOV), drugs, fraud, and traffic. We exclude arson, handling stolen goods, homicide, liquor-related (does not include public drunkenness), prostitution, trespassing, and weapons-related offences as the counts for most of these offence types were consistently quite low or not always clearly identified as criminal.

## Methods

We analyse slightly more than 2 years of days (113 weeks), controlling for the longer-term and seasonal trends. We use weekly counts to maximise the number of observations during the short time horizon for this global pandemic, while minimising volatility. As an additional method to address the volatility in weekly offence data we use a data smoothing method, the Hodrick and Prescott (1997) filter, to obtain the trend in the data for analysis. The HP (1997) filter was developed in the macroeconomics literature to identify business cycles and separates the trend, cyclical, and error components of a time series:

1 $$y_{t} = \tau_{t} + c_{t} + \in_{t}$$

where *y*_*t*_ is the time series of interest, *τ*_*t*_ is the trend component, *c*_*t*_ is the cyclical component (weekly pattern, for example), and *ϵ*_*t*_ is the error component. The trend component, *τ*_*t*_, is identified using the following function:

2 $$\mathop {\hbox{min} }\limits_{\tau } \left( {\mathop \sum \limits_{t = 1}^{T} \left( {y_{t} - \tau_{t} } \right)^{2} + \lambda \mathop \sum \limits_{t = 2}^{T - 1} \left[ {\left( {\tau_{t + 1} - \tau_{t} } \right) - \left( {\tau_{t} - \tau_{t - 1} } \right)} \right]^{2} } \right).$$

The first term in Eq. 2 is the sum of squared deviations of the original weekly time series and its trend; the second term is the sum of squares of the squared second differences, penalizing variations in the growth rate of *τ*_*t*_. All HP filter calculations are undertaken in R using the mFilter library, developed by Balcilar (2018).

Our choice to use the HP (1997) filter stems from the fact that it identifies the trend in the data without the loss of observations that occurs when using more traditional methods such as moving average calculations. It is important to note that the HP (1997) filter has its critiques. In particular, the identification and analysis of the (business) cycles in time series have been identified as potentially problematic (Hamilton 2018). However, we are using the HP ((1997)) filter to smooth a volatile (weekly) data set, not analyse the (timed) cyclical component of the data.

The primary statistical methodology is a structural break test with robust (heteroskedastic and autocorrelation consistent) standard errors. These analyses are becoming increasingly common in the criminological literature (Andresen et al. 2019; Piehl et al. 2003; Reid and Andresen 2014; Hodgkinson et al. 2018), including an analysis of COVID-19 and crime (Hodgkinson and Andresen 2020). We use a version of the Chow (1960) test to exogenously identify (we impose the break points) a change in the trends of the offence types:

3 $$\tau_{t} = \alpha + \beta_{1} Week + \beta_{2} Week^{2} + \beta_{3} OverallTrend + \gamma_{1} Lockdown + \gamma_{2} LockdownTrend + \gamma_{3} Stage + \gamma_{4} StageTrend .$$

We account for the known seasonal component in offence data (Andresen and Hodgkinson 2018; Breetzke and Cohn 2012; Cohn and Rotton 2000; Farrell and Pease 1994; Linning et al. 2017; McDowall et al. 2012) including both week and week-squared variables. *Week* is measured as sequential values (1, 2, 3, …, 52) over the course of a year, whereas *Week*^*2*^ is the squared value of *Week*—these two variables account for the known seasonal effect in the data, not the week-to-week volatility filtered out using the HP (1997) filter. *Overall Trend*, measured as sequential values for the entire time series (1, 2, 3, …, 113), captures any underlying trend in the data for the entire time period.

We include two break points in the data and test for their statistical significance. The first breakpoint captures the lockdown, both its immediate effect (if any) and any change in trend; this break point is 25 March 2020. The second breakpoint is the staged relaxation of social restrictions (beginning with the opening of restaurants, pubs, and bars), both its immediate effect (if any) and any change in trend; this break point is 16 May 2020. The Queensland government implemented a number of relaxations to social restrictions, but we chose this date because it represented the opening of restaurants, pubs, and bars that necessarily increases movements outside of the home and work environments. Each break-based variable has the value of zero before its representative break time and unity (*Lockdown* and *Stage*) or sequential values (*LockdownTrend* and *StageTrend*) thereafter. All estimation for the sequential Chow tests is undertaken using R: A language and environment for statistical computing, version 3.5.3 (R Core Team 2019).

## Results

The results of the structural break tests are presented in Tables 1, 2, 3, 4, 5, 6, 7, 8, 9, 10, 11 and 12. In addition to the structural break results, we include the pre- and post-lockdown average weekly counts by offence type and QPS district, for context regarding the magnitude of the various parameters. For the structural breaks we present the overall trend variable, the lockdown dummy variable (immediate impact of lockdown), the lockdown trend variable (change in trend after lockdown), the stages dummy variable (immediate impact of staged reduction in social restrictions), and the stages trend variable (change in trend after staged reduction in social restrictions).

**Table 1:** Structural break analysis results, good order

	Pre-lockdown average	Post-lockdown average	Overall trend	Lockdown	Lockdown trend	Stages	Stages trend	Adjusted-*R*^2^
Capricornia	58.16	39.53**	− 0.054**	− 7.418**	0.545**	2.004**	− 0.349**	0.866
Darling Downs	51.29	28.27**	− 0.137**	− 8.375**	− 0.126	1.764**	− 0.361	0.886
Far North	86.43	66.53**	0.099**	− 11.316**	− 2.004**	3.067	1.657**	0.835
Gold Coast	81.70	42.53**	− 0.261**	− 19.104**	− 0.683	9.261**	0.416	0.861
Ipswich	51.34	20.73**	− 0.199**	1.119	− 2.401**	− 2.348**	1.559**	0.935
Logan	60.35	41.60**	− 0.209**	0.898	− 0.870**	1.912**	− 0.356	0.777
Mackay	41.65	26.73**	− 0.063**	− 4.279**	− 0.253	1.542**	− 0.744*	0.825
Moreton	43.15	27.27**	− 0.016	− 7.638**	− 1.388**	0.747	1.713**	0.883
Mount Isa	22.48	12.93**	− 0.127**	1.773	− 0.248	− 0.533**	− 0.063	0.789
North Brisbane	146.86	103.40**	− 0.062	− 24.076**	− 1.222	14.371**	− 2.453**	0.869
South Brisbane	83.39	55.27**	− 0.143**	− 13.014**	− 1.153**	5.388**	2.416**	0.903
South West	29.26	21.13**	0.005	− 4.091**	− 0.141	2.169**	− 1.301	0.792
Sunshine Coast	48.33	31.73**	− 0.071*	− 12.639**	− 0.107	3.747**	− 0.519	0.701
Townsville	63.63	47.00**	− 0.005	− 12.568**	− 0.784*	− 0.226	2.034**	0.797
Wide Bay Burnett	41.85	30.93**	0.047	− 15.517**	0.014	6.926**	− 0.594	0.568

* 10% significance level; ** 5% significance level

**Table 2:** Structural break analysis results, mischief

	Pre-lockdown average	Post-lockdown average	Overall trend	Lockdown	Lockdown trend	Stages	Stages trend	Adjusted-*R*^2^
Capricornia	37.50	27.33**	− 0.000	− 10.175**	− 0.347	4.749**	1.171*	0.564
Darling Downs	30.50	22.53**	0.003	− 6.082**	0.113	3.308**	− 0.826*	0.601
Far North	58.83	44.93**	0.174**	− 14.664**	− 1.310**	0.746	1.633**	0.702
Gold Coast	78.55	60.53**	− 0.021	− 9.022**	− 0.589	2.326**	0.694*	0.842
Ipswich	37.03	14.27**	− 0.005	− 5.101*	− 2.047**	− 3.224**	1.131**	0.905
Logan	55.89	44.20**	0.009	1.934	− 1.817**	2.819**	1.079**	0.776
Mackay	21.32	18.13**	0.071**	− 1.432	− 0.592**	− 0.618	0.131	0.531
Moreton	37.87	27.20**	0.017	− 8.425**	− 0.394	− 0.114	0.688**	0.589
Mount Isa	18.51	13.47**	0.035	− 5.205**	0.022	2.517**	− 0.543	0.551
North Brisbane	80.40	60.33**	0.044	− 8.067**	− 1.595**	7.698**	− 1.276**	0.873
South Brisbane	79.74	58.67**	− 0.136**	− 13.582**	0.509	7.734**	− 1.383	0.863
South West	19.01	14.20**	− 0.009	− 0.232	− 0.520**	− 0.524	0.914**	0.692
Sunshine Coast	34.42	24.73**	− 0.024	− 6.101**	− 0.301	1.338	0.179	0.554
Townsville	46.20	29.53**	0.006	− 10.065**	− 1.363**	− 1.432	2.361**	0.643
Wide Bay Burnett	34.96	31.00**	− 0.005	− 3.903	0.399*	2.801**	− 1.718**	0.358

* 10% significance level; ** 5% significance level

**Table 3:** Structural break analysis results, assault

	Pre-lockdown average	Post-lockdown average	Overall trend	Lockdown	Lockdown trend	Stages	Stages trend	Adjusted-*R*^2^
Capricornia	24.48	19.27**	− 0.035	− 4.741**	0.304	1.190	0.117	0.547
Darling Downs	17.30	13.60**	0.002	− 3.518**	0.054	2.766**	− 0.041	0.625
Far North	46.99	41.47**	0.118**	− 4.914**	− 0.989**	− 0.395	1.919**	0.605
Gold Coast	60.60	49.47**	0.068**	− 7.421**	− 0.036	5.083**	− 1.826**	0.812
Ipswich	17.76	6.67**	0.008	− 4.233**	− 0.790**	− 0.531*	0.139	0.902
Logan	27.57	23.27**	− 0.009	− 1.334	− 0.403*	0.484	1.309**	0.598
Mackay	13.82	13.00	0.026	3.713*	− 1.009**	− 0.496	1.621**	0.250
Moreton	20.06	16.33**	− 0.001	− 2.706**	0.463**	1.470**	− 1.873**	0.447
Mount Isa	18.50	13.00**	− 0.006	− 6.044**	0.0507*	0.625	− 0.212	0.589
North Brisbane	44.53	32.47**	0.016	− 5.694**	− 0.729**	3.595**	0.868**	0.845
South Brisbane	38.00	30.87**	− 0.097**	− 5.537**	0.453	2.689**	0.362	0.528
South West	13.31	14.20**	0.016**	− 0.391	− 0.432**	− 0.581**	1.024**	0.627
Sunshine Coast	15.79	12.27**	− 0.001	− 3.039**	− 0.015	1.957**	− 0.373**	0.469
Townsville	36.53	32.20*	− 0.010	− 1.191	0.239	1.083*	− 1.479**	0.437
Wide Bay Burnett	19.83	17.40	0.052**	− 3.644**	− 0.451	2.632**	0.892**	0.583

* 10% significance level; ** 5% significance level

**Table 4:** Structural break analysis results, robbery

	Pre-lockdown average	Post-lockdown average	Overall trend	Lockdown	Lockdown trend	Stages	Stages trend	Adjusted-*R*^2^
Capricornia	1.50	0.67**	0.014**	− 0.479*	− 0.206**	0.127*	0.280**	0.537
Darling Downs	1.24	1.07	0.015**	− 0.771**	− 0.024	− 0.575**	0.129**	0.590
Far North	2.36	2.00	0.021**	0.446	− 0.222**	− 0.242*	0.179*	0.468
Gold Coast	5.15	3.67**	0.018**	− 2.754**	− 0.083	0.659*	− 0.082	0.453
Ipswich	2.31	0.80**	0.004*	− 1.243**	− 0.068	0.126	0.057	0.816
Logan	5.12	3.33**	− 0.011	− 2.322**	0.198*	0.928**	− 0.249*	0.530
Mackay	0.71	0.67	0.002**	0.648**	− 0.129**	0.013	0.184**	0.050
Moreton	2.48	1.87	0.017**	− 0.746**	− 0.144**	0.099	0.006	0.486
Mount Isa	0.27	0.27	0.001	0.323*	− 0.046**	0.036	0.015	0.214
North Brisbane	6.43	4.20**	0.022**	− 1.718**	− 0.295**	0.250	0.324**	0.372
South Brisbane	7.24	5.33**	0.034**	0.054	− 0.668**	0.911**	0.649**	0.584
South West	0.50	0.13**	− 0.002**	− 0.104*	0.011	0.001	− 0.009	0.320
Sunshine Coast	1.51	1.07	− 0.003*	0.232	− 0.048	− 0.036	− 0.015	0.103
Townsville	2.65	2.27	0.011**	− 0.972	− 0.129**	0.221	0.304**	0.163
Wide Bay Burnett	1.49	1.33	0.008**	− 0.565**	− 0.049	− 0.030	0.126*	0.255

* 10% significance level; ** 5% significance level

**Table 5:** Structural break analysis results, other violence

	Pre-lockdown average	Post-lockdown average	Overall trend	Lockdown	Lockdown trend	Stages	Stages trend	Adjusted-*R*^2^
Capricornia	3.87	5.33*	− 0.014**	1.448**	0.176**	0.482**	− 0.553**	0.393
Darling Downs	2.55	2.47	− 0.013**	− 0.183	0.196**	0.537**	− 0.239**	0.367
Far North	4.68	5.27	0.001	0.235	0.259**	− 0.311**	− 0.488**	0.389
Gold Coast	10.23	9.07	− 0.033**	− 2.008**	0.578**	2.738**	− 0.846**	0.546
Ipswich	3.32	1.40**	0.009*	− 1.050**	− 0.252**	− 0.077	0.187**	0.713
Logan	5.53	5.87	− 0.022**	− 1.542	0.373**	1.405**	− 0.495**	0.434
Mackay	1.78	1.87	0.002	− 0.590**	− 0.051	0.166	0.469**	0.389
Moreton	4.68	3.73	− 0.023**	− 0.413	0.291**	0.907**	− 0.784**	0.445
Mount Isa	1.61	2.00	0.004	0.188*	0.049**	0.639**	− 0.246**	0.366
North Brisbane	8.30	7.53	0.002	1.092*	0.124	0.696**	− 1.092**	0.455
South Brisbane	6.93	5.00**	− 0.041**	− 0.412	0.222**	− 0.039	0.114	0.599
South West	2.50	2.40	− 0.017**	0.655**	0.001	− 0.739**	0.176*	0.352
Sunshine Coast	3.67	3.40	− 0.019**	− 1.120**	0.435**	0.850**	− 0.756**	0.504
Townsville	4.88	3.07**	− 0.031**	− 1.968**	0.451**	1.141**	-0.955**	0.609
Wide Bay Burnett	3.87	3.27	− 0.006	− 0.670	0.168**	− 0.092	− 0.152**	0.495

* 10% significance level; ** 5% significance level

**Table 6:** Structural break analysis results, total violence

	Pre-lockdown average	Post-lockdown average	Overall trend	Lockdown	Lockdown trend	Stages	Stages trend	Adjusted-*R*^2^
Capricornia	29.85	25.27**	− 0.035	− 3.773*	0.275	1.799*	− 0.155	0.428
Darling Downs	21.09	17.13**	0.001	− 4.472**	0.226	2.728**	− 0.151	0.594
Far North	54.03	48.73*	0.140**	− 4.233**	− 0.953**	− 0.945**	1.611**	0.619
Gold Coast	75.99	62.20**	0.054	− 12.183**	0.459	8.480**	− 2.753**	0.783
Ipswich	23.38	8.87**	0.021	− 6.527**	− 1.111**	− 0.474	0.383*	0.919
Logan	38.22	32.47**	− 0.042**	− 5.198*	0.169	2.816**	0.566	0.638
Mackay	16.31	15.53	0.029	3.778*	− 1.192**	− 0.313	2.275**	0.319
Moreton	27.22	21.93**	− 0.008	− 3.869**	0.609**	2.475**	− 2.650**	0.492
Mount Isa	20.38	15.27**	− 0.001	− 5.532**	0.510**	1.311*	− 0.443	0.579
North Brisbane	59.26	44.20**	0.040	− 6.322**	− 0.900*	4.546**	0.099	0.759
South Brisbane	52.17	41.20**	− 0.103**	− 5.889**	0.006	3.557**	1.128**	0.578
South West	16.31	13.93*	− 0.003	0.163	− 0.420**	− 1.317**	1.193**	0.604
Sunshine Coast	20.97	16.73**	− 0.024*	− 3.922**	0.371**	2.771**	− 1.142**	0.529
Townsville	44.06	37.53**	− 0.029	− 4.136**	0.561	2.449**	− 2.131**	0.478
Wide Bay Burnett	25.18	22.00*	0.055**	− 4.887**	− 0.330	2.503**	0.865**	0.635

* 10% significance level; ** 5% significance level

**Table 7:** Structural break analysis results, burglary

	Pre-lockdown average	Post-lockdown average	Overall trend	Lockdown	Lockdown trend	Stages	Stages trend	Adjusted-*R*^2^
Capricornia	38.62	25.47**	0.086**	− 9.879**	− 2.143**	4.269**	2.002**	0.742
Darling Downs	37.89	27.13**	0.098**	− 6.805**	− 2.053**	5.946**	1.767**	0.469
Far North	71.38	42.13**	0.327**	− 31.535**	− 3.921**	8.871**	3.052**	0.715
Gold Coast	76.45	73.07	0.139*	21.023**	− 5.166**	4.889**	1.826**	0.715
Ipswich	36.72	11.73**	0.109**	− 9.266**	− 2.842**	− 1.009	1.841**	0.922
Logan	74.21	55.20**	0.199**	− 7.223**	− 4.103**	3.078**	3.694**	0.841
Mackay	28.23	15.80**	0.006	− 5.086	− 1.069**	1.298**	0.964	0.548
Moreton	38.80	31.00**	0.069**	− 4.853	− 0.135	2.386**	− 2.146**	0.668
Mount Isa	15.29	9.80**	0.054	− 3.611	− 0.719*	0.609	0.903**	0.533
North Brisbane	106.96	78.13**	0.584**	− 12.223	− 6.447**	8.399**	1.639**	0.886
South Brisbane	122.21	86.27**	0.184*	− 4.108	− 6.652**	10.324**	4.664**	0.655
South West	18.90	13.47**	0.015*	− 3.265**	− 0.508**	2.704**	− 0.039	0.675
Sunshine Coast	31.24	25.00**	0.042*	6.021**	− 3.394**	0.055	4.879**	0.683
Townsville	67.93	50.87**	0.336**	2.615	− 7.909**	15.071**	6.798**	0.402
Wide Bay Burnett	31.34	21.67**	0.032	− 7.351**	− 0.966*	3.371**	0.858*	0.703

* 10% significance level; ** 5% significance level

**Table 8:** Structural break analysis results, theft

	Pre-lockdown average	Post-lockdown average	Overall trend	Lockdown	Lockdown trend	Stages	Stages trend	Adjusted-*R*^2^
Capricornia	92.44	56.53**	0.207**	− 10.389**	− 5.710**	1.581	4.088**	0.799
Darling Downs	91.86	57.53**	0.078	− 16.572**	− 4.082**	3.533**	4.175**	0.817
Far North	138.38	75.53**	0.349**	− 37.605**	− 8.427**	6.241*	8.219**	0.887
Gold Coast	365.94	231.53**	0.314	− 57.703**	− 14.052**	29.968**	4.956*	0.854
Ipswich	143.26	42.53**	0.085	− 33.663**	− 10.011**	− 3.563**	8.216**	0.968
Logan	231.09	136.13**	0.672**	− 51.044**	-12.535**	12.159**	8.902**	0.904
Mackay	70.23	42.60**	0.173**	− 16.752**	− 2.883**	3.321*	3.089**	0.729
Moreton	147.49	81.87**	0.277**	− 52.921**	− 6.664**	8.974**	6.051**	0.856
Mount Isa	23.97	14.53**	0.176**	− 10.787**	− 1.177**	0.286	0.866**	0.729
North Brisbane	420.48	265.87**	1.555**	− 78.138**	− 27.490**	14.962**	25.981**	0.949
South Brisbane	357.14	254.87**	0.968**	− 44.203**	− 17.509**	22.584**	10.098**	0.928
South West	35.98	24.40**	0.068**	− 8.797**	− 1.274**	2.143**	1.372**	0.723
Sunshine Coast	128.95	81.47**	0.115	− 12.838	− 6.765**	7.993**	5.833**	0.663
Townsville	118.50	56.13**	0.323**	− 44.289**	− 6.659**	9.197**	4.941**	0.858
Wide Bay Burnett	99.36	61.40**	0.059	− 30.089**	− 1.116	7.318**	− 0.621	0.835

* 10% significance level; ** 5% significance level

**Table 9:** Structural break analysis results, theft of vehicle

	Pre-lockdown average	Post-lockdown average	Overall trend	Lockdown	Lockdown trend	Stages	Stages trend	Adjusted-*R*^2^
Capricornia	9.95	8.93	0.067**	− 2.696**	− 0.526**	1.849**	0.296	0.594
Darling Downs	11.70	9.80	− 0.029**	− 0.385	− 0.061	1.238**	− 0.036	0.250
Far North	18.60	13.53**	0.095**	− 8.745**	− 0.829**	4.149**	0.607	0.732
Gold Coast	44.24	37.60**	0.111**	0.499	− 2.419**	3.157**	1.350**	0.714
Ipswich	14.63	4.93**	0.037**	− 4.602**	− 0.899**	− 0.641*	0.537*	0.790
Logan	32.02	26.60**	0.111**	− 3.399*	− 1.286**	1.855**	0.922*	0.745
Mackay	9.66	7.67**	0.064**	− 1.944	− 0.538**	1.095**	0.091	0.611
Moreton	16.63	12.47**	0.048**	− 5.108**	− 0.559**	2.536**	− 0.374	0.651
Mount Isa	3.20	1.60**	0.022**	− 2.300**	− 0.052	0.481*	0.044	0.579
North Brisbane	39.82	33.73**	0.205**	− 8.453**	− 1.774**	2.511**	1.683**	0.829
South Brisbane	41.49	33.53**	0.094**	− 1.003	− 1.859**	1.143	1.632**	0.619
South West	4.96	4.73	− 0.001	− 0.268	− 0.149**	0.902**	0.105*	0.216
Sunshine Coast	13.70	11.93	− 0.001	1.849	− 1.141**	0.257	1.429**	0.639
Townsville	19.45	13.13**	0.077*	− 3.478	− 1.455**	4.205**	0.885*	0.462
Wide Bay Burnett	9.15	7.87*	0.001	− 0.284	− 0.193**	0.714**	0.421**	0.183

* 10% significance level; ** 5% significance level

**Table 10:** Structural break analysis results, drugs

	Pre-lockdown average	Post-lockdown average	Overall trend	Lockdown	Lockdown trend	Stages	Stages trend	Adjusted-*R*^2^
Capricornia	63.00	62.53	− 0.012	7.592**	1.018**	− 2.773	− 5.519**	0.344
Darling Downs	77.06	61.87**	− 0.227**	9.795**	0.103	− 2.559	− 2.484**	0.722
Far North	88.74	78.73**	− 0.067	6.739	0.282	− 0.932	− 4.233**	0.494
Gold Coast	133.07	107.60**	0.076	− 0.917	− 2.996**	− 4.319	0.859	0.458
Ipswich	58.37	26.13**	− 0.092**	5.959**	− 3.223**	− 6.292**	0.174	0.913
Logan	86.48	97.40**	− 0.009	28.078**	− 2.572**	− 0.230	0.573	0.587
Mackay	52.27	61.73**	0.036	16.866**	− 1.135	− 3.239	− 0.279	0.409
Moreton	74.13	66.33*	− 0.055	2.251	− 0.147	− 0.847	− 2.379**	0.496
Mount Isa	13.24	13.33	− 0.006	8.023**	− 0.866**	− 1.426	0.451	0.557
North Brisbane	188.42	195.07	0.195**	3.360	2.685**	5.779**	− 12.564**	0.455
South Brisbane	124.48	119.40	− 0.117	21.587**	− 0.537	− 2.625	− 6.262**	0.542
South West	41.50	41.33	0.053	5.844**	− 0.570	− 1.034	− 1.570**	0.186
Sunshine Coast	71.53	62.27*	− 0.047	12.183**	− 1.711**	− 4.496**	− 2.015**	0.564
Townsville	76.43	78.67	0.091**	0.117	− 0.291	1.173	− 3.334**	0.146
Wide Bay Burnett	62.30	51.93**	0.013	− 1.833	− 0.948*	5.009**	− 1.007	0.305

* 10% significance level; ** 5% significance level

**Table 11:** Structural break analysis results, fraud

	Pre-lockdown average	Post-lockdown average	Overall trend	Lockdown	Lockdown trend	Stages	Stages trend	Adjusted-*R*^2^
Capricornia	13.09	6.73**	0.028**	− 2.367	− 0.189	− 0.732	− 0.851**	0.454
Darling Downs	14.67	6.33**	− 0.012	− 5.907**	0.051	0.656	− 0.292	0.592
Far North	18.39	12.40**	− 0.049**	3.140	− 0.237	− 1.527**	− 1.418**	0.603
Gold Coast	53.37	29.13**	− 0.143**	− 14.184**	− 0.351	7.151**	− 0.951	0.789
Ipswich	17.66	4.07**	0.002	− 0.884	− 1.675**	− 1.712**	1.242**	0.760
Logan	30.37	19.80**	0.071**	− 5.368*	− 1.079**	2.594**	0.474	0.626
Mackay	9.70	4.00**	0.043*	− 1.876*	− 0.136	− 1.348**	− 0.939*	0.494
Moreton	20.23	11.93**	− 0.044*	− 6.012**	0.324	2.714**	− 0.913*	0.475
Mount Isa	1.93	0.40**	0.018**	− 0.698**	− 0.252**	− 0.092	0.166**	0.522
North Brisbane	62.02	34.73**	− 0.050	− 11.786**	− 2.379**	4.534**	2.220**	0.767
South Brisbane	53.02	36.60**	− 0.004	− 4.621	− 1.148**	− 0.282	− 0.001	0.639
South West	4.13	3.13	0.022**	− 2.321**	0.216*	0.659**	− 1.118**	0.393
Sunshine Coast	16.94	9.13**	− 0.058**	− 1.948	− 0.702	3.569**	0.261	0.446
Townsville	12.84	4.20**	− 0.005	− 1.882	− 1.289**	3.212**	1.069**	0.668
Wide Bay Burnett	14.12	10.07	− 0.025	− 4.579*	0.563**	0.622	0.034	0.478

* 10% significance level; ** 5% significance level

**Table 12:** Structural break analysis results, traffic

	Pre-lockdown average	Post-lockdown average	Overall trend	Lockdown	Lockdown trend	Stages	Stages trend	Adjusted-*R*^2^
Capricornia	45.38	40.67*	− 0.004	− 7.306	0.324	1.180	− 0.113	0.311
Darling Downs	60.36	41.87**	− 0.125**	− 9.075**	0.392	− 1.889**	0.934	0.635
Far North	76.83	62.67**	− 0.077	− 18.339**	0.983	7.279**	− 0.929	0.449
Gold Coast	86.68	57.20**	− 0.185**	− 6.271**	− 1.244**	2.626*	0.426	0.862
Ipswich	53.72	18.47**	− 0.132**	− 1.265	− 2.445**	− 4.305**	0.659	0.893
Logan	54.36	43.33**	− 0.058*	− 2.959*	− 1.454**	− 0.166	2.892**	0.692
Mackay	39.55	36.07	− 0.049**	− 7.279**	2.068**	6.748**	− 4.420**	0.626
Moreton	44.95	33.07**	− 0.036	− 15.394**	0.450	1.109	1.241**	0.494
Mount Isa	11.67	10.33	− 0.032**	0.275	0.125	− 1.082**	0.815**	0.638
North Brisbane	77.67	66.40**	− 0.029	− 6.288*	− 0.343	7.272**	− 0.938	0.359
South Brisbane	82.37	55.60**	− 0.174**	− 21.275**	− 0.210	3.672**	1.599**	0.773
South West	36.71	25.87**	− 0.008	− 10.241**	− 0.904**	2.624**	2.499**	0.599
Sunshine Coast	59.83	52.33**	− 0.075**	0.704	− 0.112	− 3.515**	0.099	0.371
Townsville	41.17	42.87	− 0.027**	1.243	− 0.181	− 2.280**	0.988**	0.420
Wide Bay Burnett	42.16	30.53**	0.006	− 14.845**	1.041**	0.235	− 0.295	0.616

* 10% significance level; ** 5% significance level

**Fig. 4:**
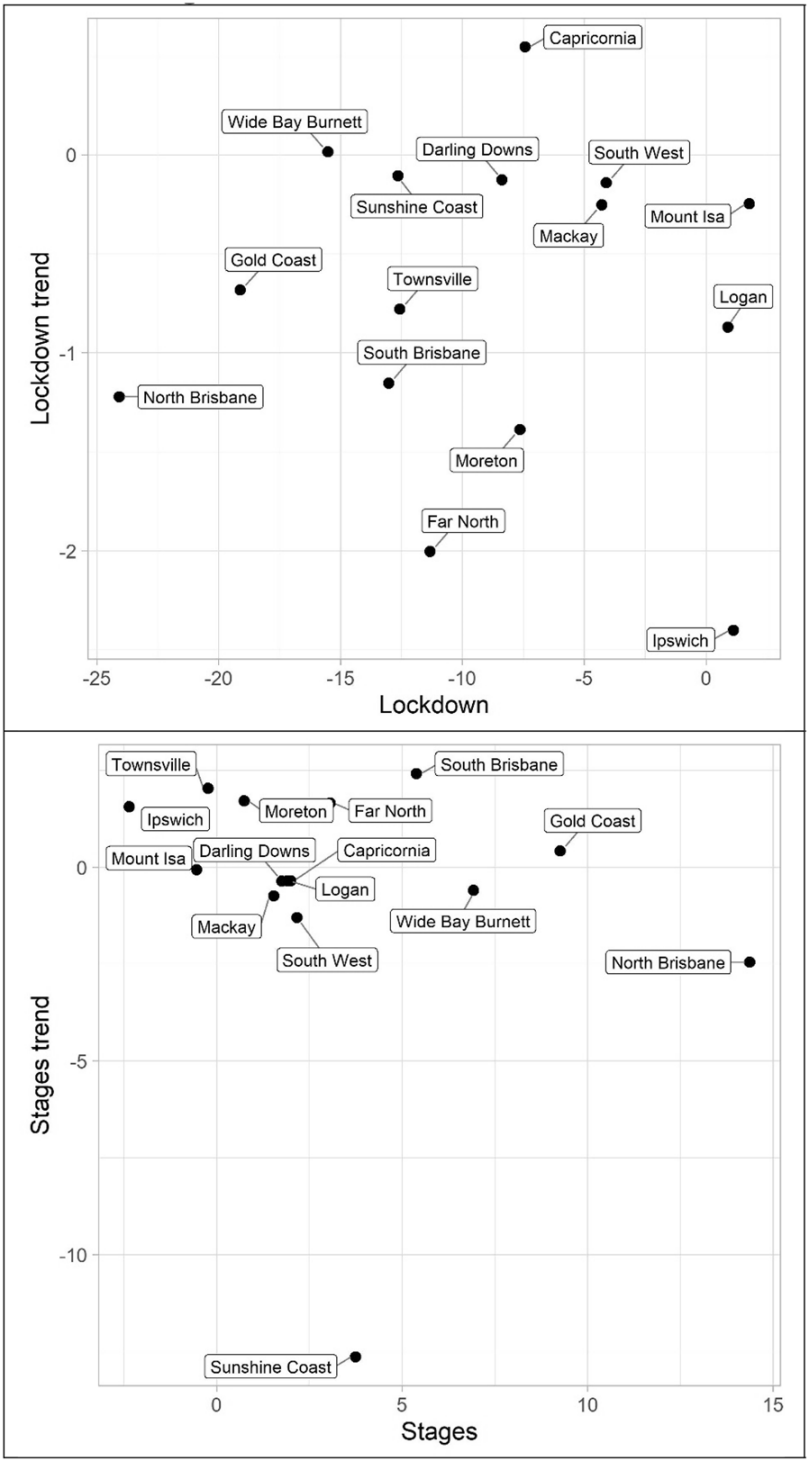
Structural break parameter plots, lockdown and staged relaxation of social restrictions, good order

**Fig. 5:**
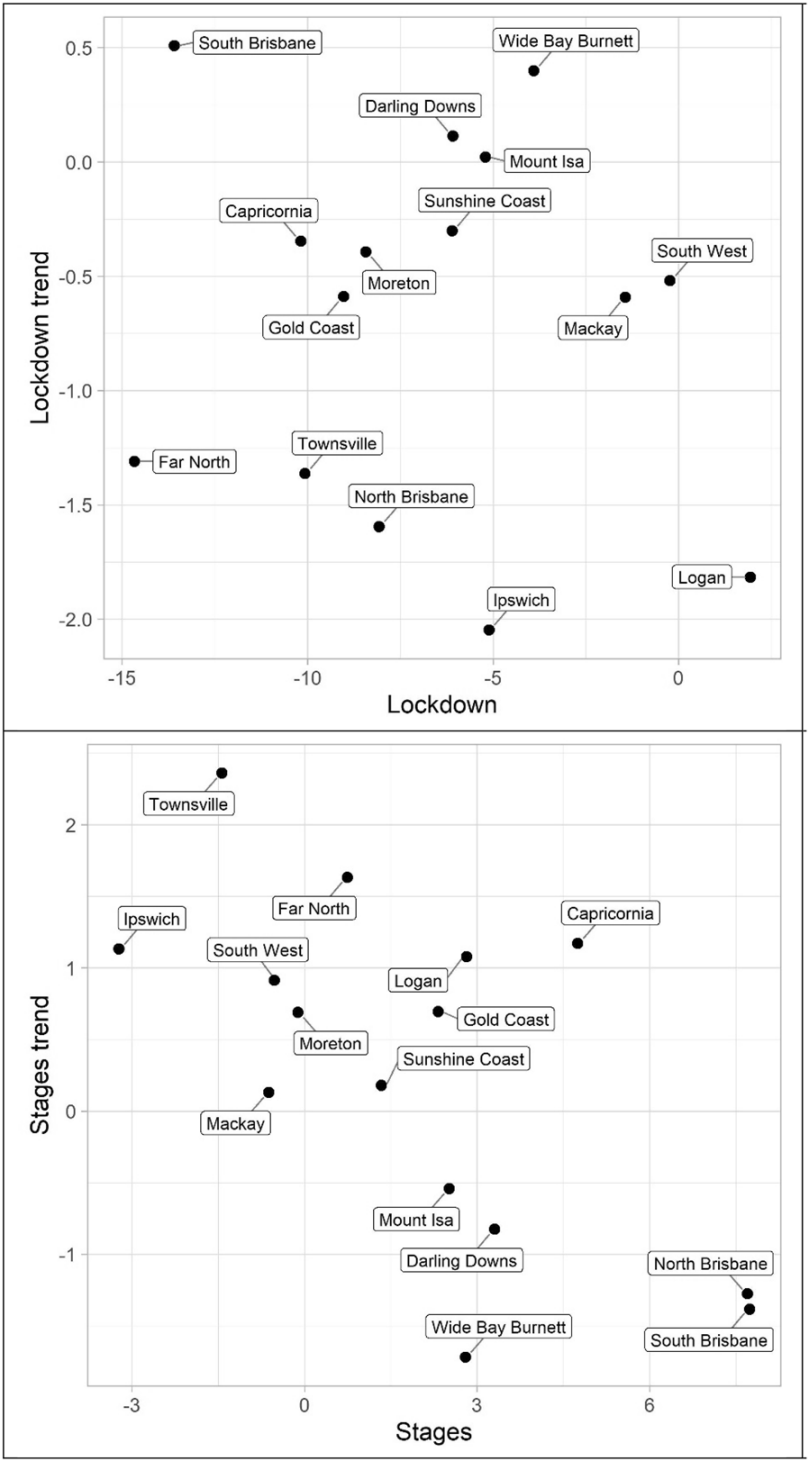
Structural break parameter plots, lockdown and staged relaxation of social restrictions, mischief

The results for the social disorder offences, good order and mischief, are reported in Tables 1 and 2 and Figs. 4 and 5. The dots in the figures represent the values of the parameters for the lockdown and staged relaxation of social restrictions (the immediate changes and subsequent changes in trend)—see the tables for statistical significance. For example, in Fig. 4, South Brisbane has negative parameters during lockdown for both the immediate and trend variables, whereas it has positive parameters during the relaxation of social restrictions for the immediate and trend variables. When statistically significant, Lockdown is always negative and with the exception of Capricornia Lockdown trend is also negative, this shows a clear pattern of notable decreases, most often immediate drops, in social disorder. Given that social disorder, by definition, most often occurs in public places, these results are as expected. With regards to Stages and Stage trend, the most common result is both parameters being positive indicating a return to some social disorder once social restrictions were relaxed. However, the results here are less consistent. Some districts exhibited immediate increases in both the dummy and trend variables (e.g. Logan and South Brisbane), while others exhibited immediate increases followed by subsequent decreases in trend (e.g. Darling Downs, Mackay, and North Brisbane)—some districts did continue to have immediate drops in social disorder during the staged relaxation of social restrictions (e.g. Ipswich and Mount Isa). These differences in trends during the staged relaxation of social restrictions cannot be known based on the data but may be due to differences in policing responses across QPS districts.

**Fig. 6:**
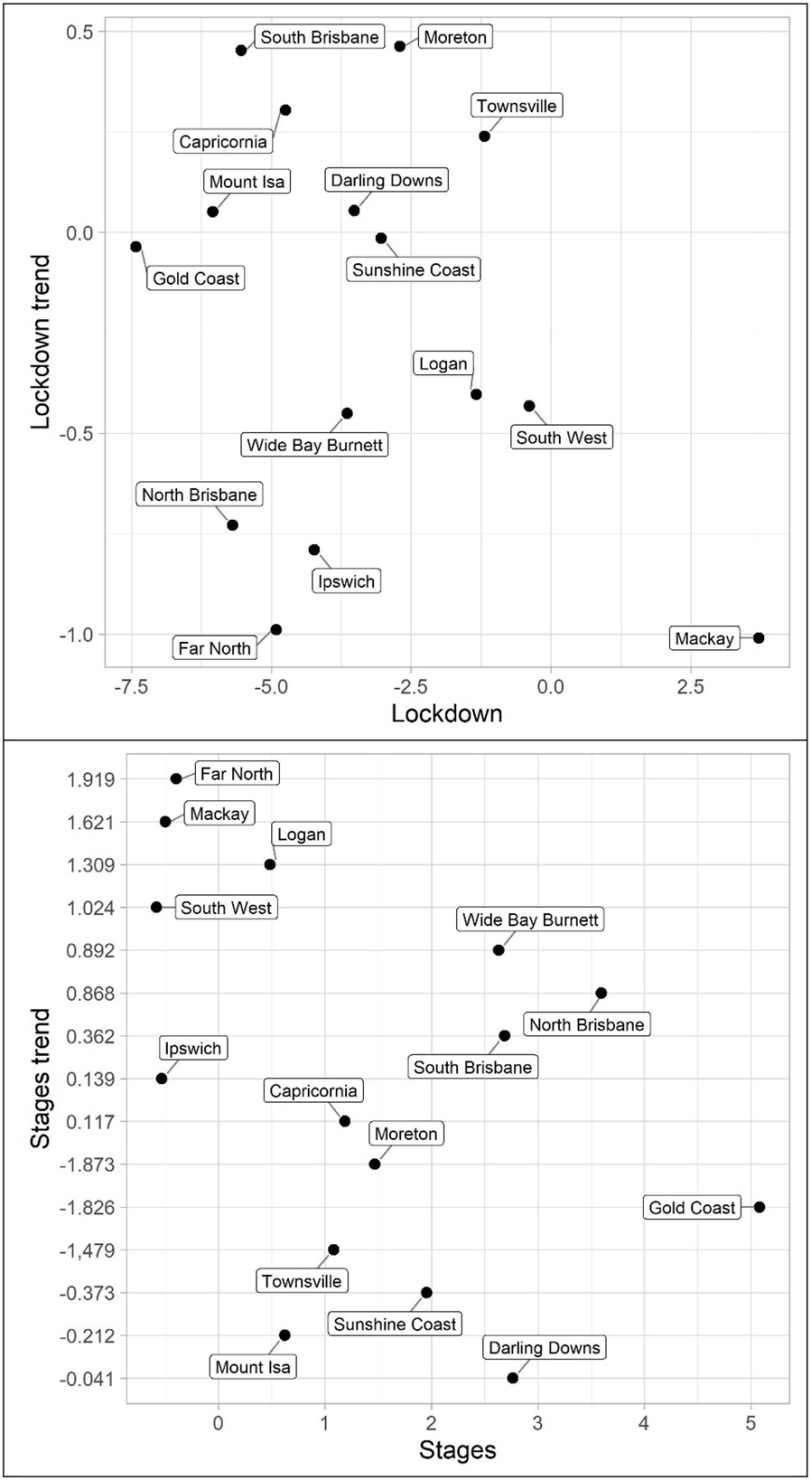
Structural break parameter plots, lockdown and staged relaxation of social restrictions, assault

**Fig. 7:**
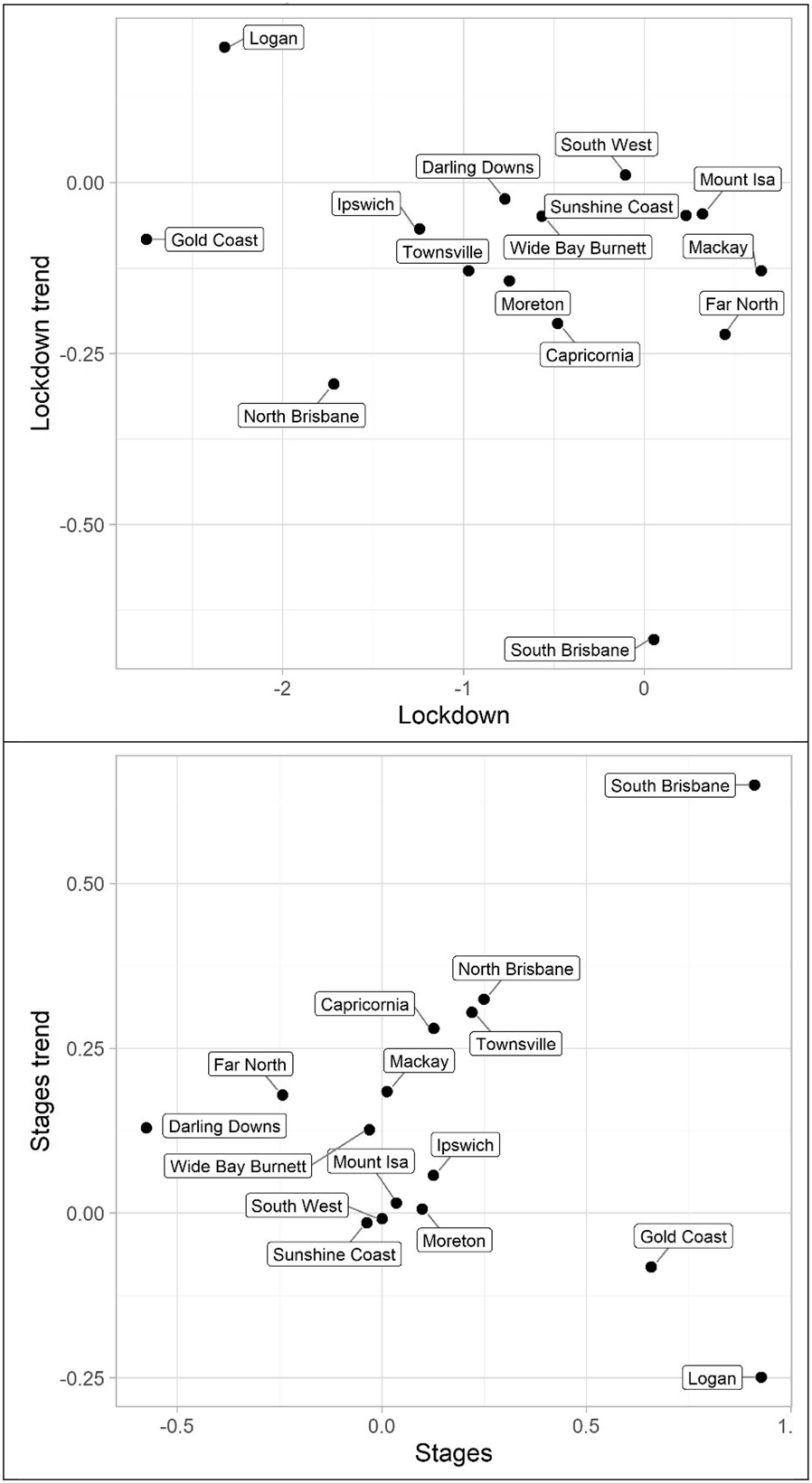
Structural break parameter plots, lockdown and staged relaxation of social restrictions, robbery

**Fig. 8:**
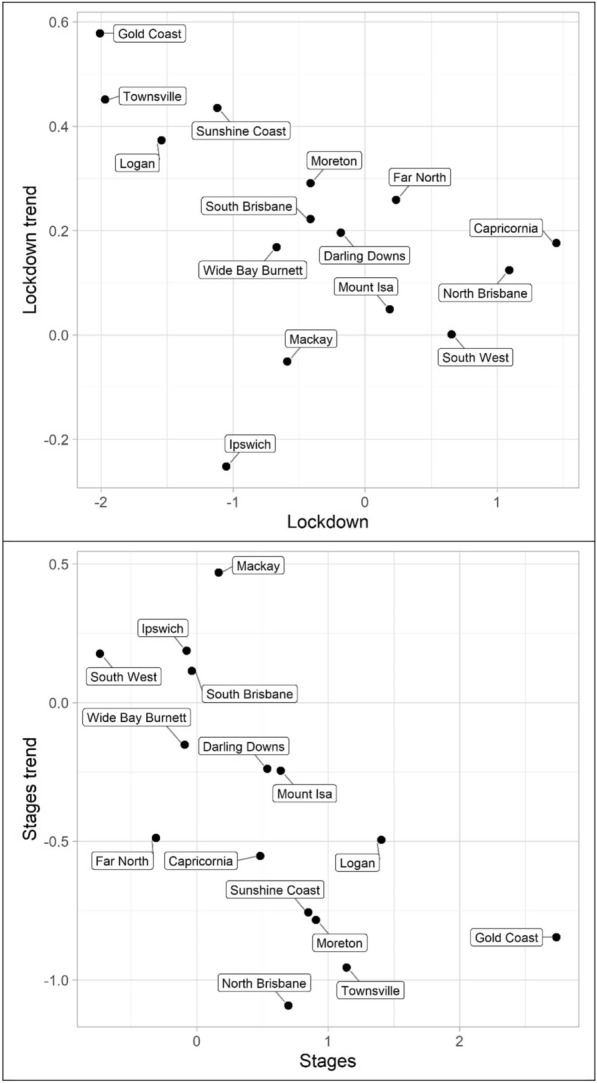
Structural break parameter plots, lockdown and staged relaxation of social restrictions, other violence

**Fig. 9:**
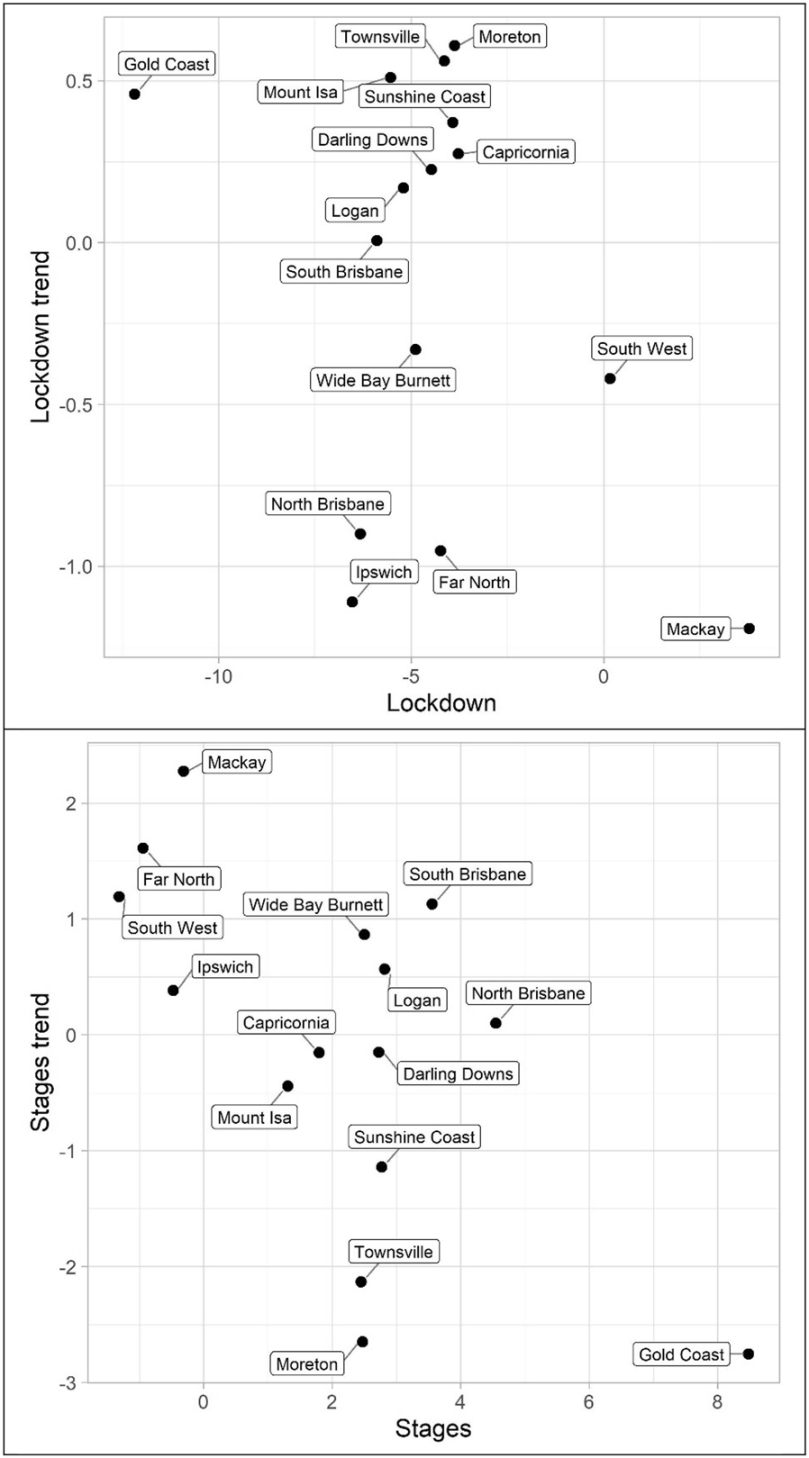
Structural break parameter plots, lockdown and staged relaxation of social restrictions, total violence

The impact of lockdown and staged relaxation of social restrictions have far more varied results with violent crime (assault, robbery, other violence, and total violence), shown in Tables 3, 4, 5 and 6 and Figs. 6, 7, 8 and 9. With regard to lockdown, assault most often exhibited the expected pattern of immediate decreases and continued decreases in trend; this is also the case for robbery. Other violence, however, is far more varied, particularly with the trend during lockdown, most often increasing. Overall, aside from Mackay, when significant there are immediate drops in total violence (Table 6) with changing trends varying from district to district; and similar to the social disorder results, the stages dummy and trend variables are not as consistent as the lockdown variables. However, in almost all cases (see Far North and Wide Bay Burnett, other violence, for exceptions) one of the two stages variables is positive when statistically significant.

**Fig. 10:**
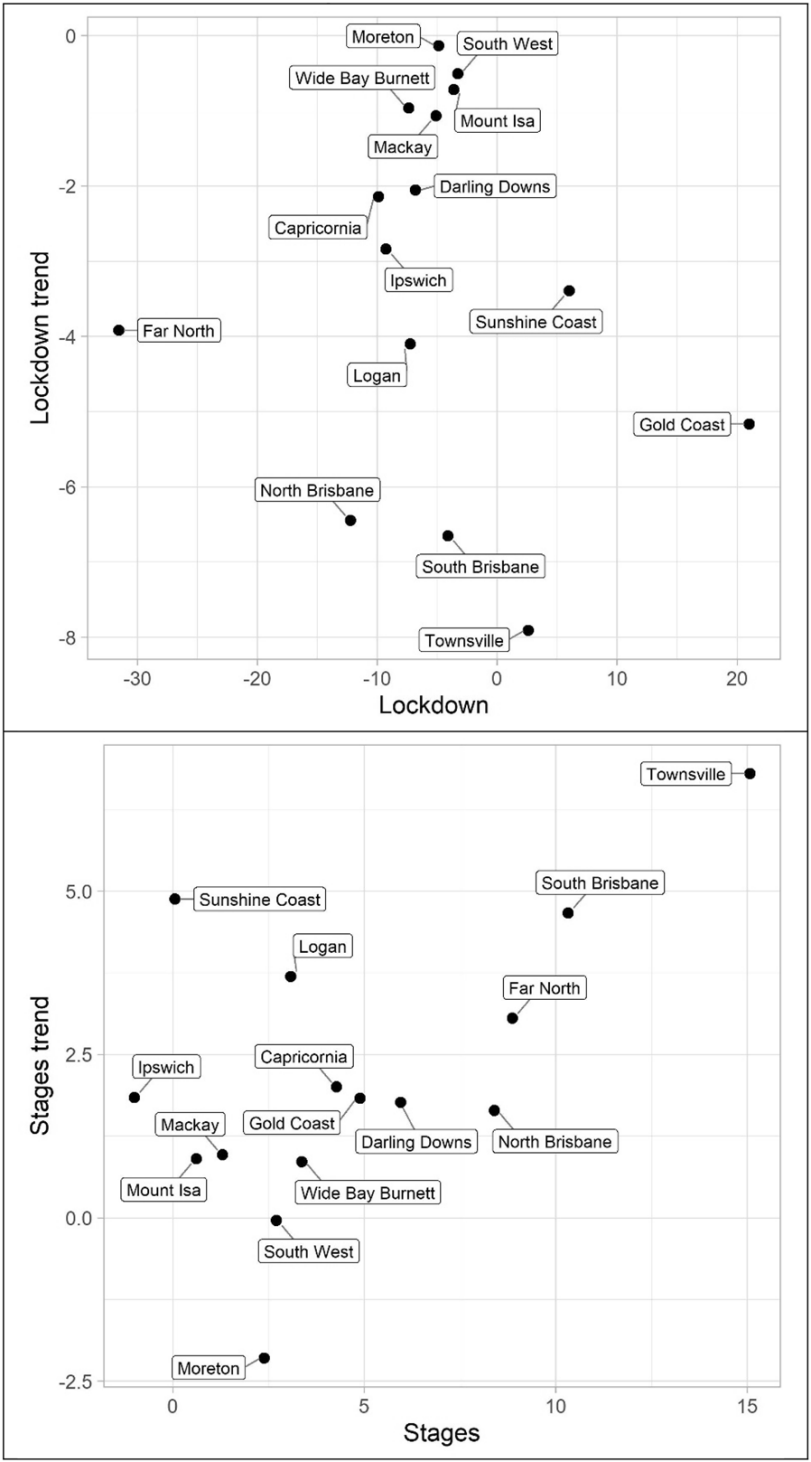
Structural break parameter plots, lockdown and staged relaxation of social restrictions, burglary

**Fig. 11:**
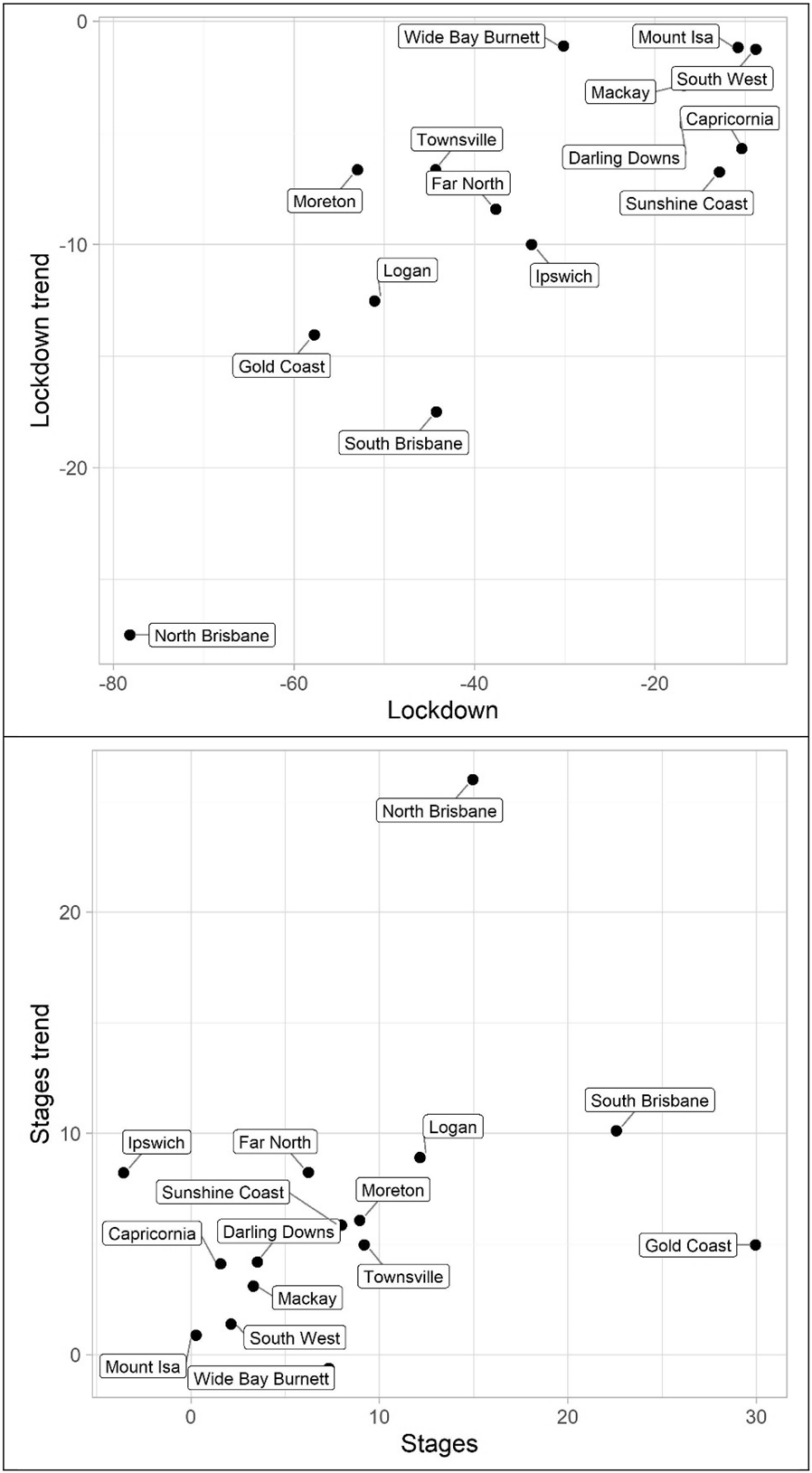
Structural break parameter plots, lockdown and staged relaxation of social restrictions, theft

**Fig. 12:**
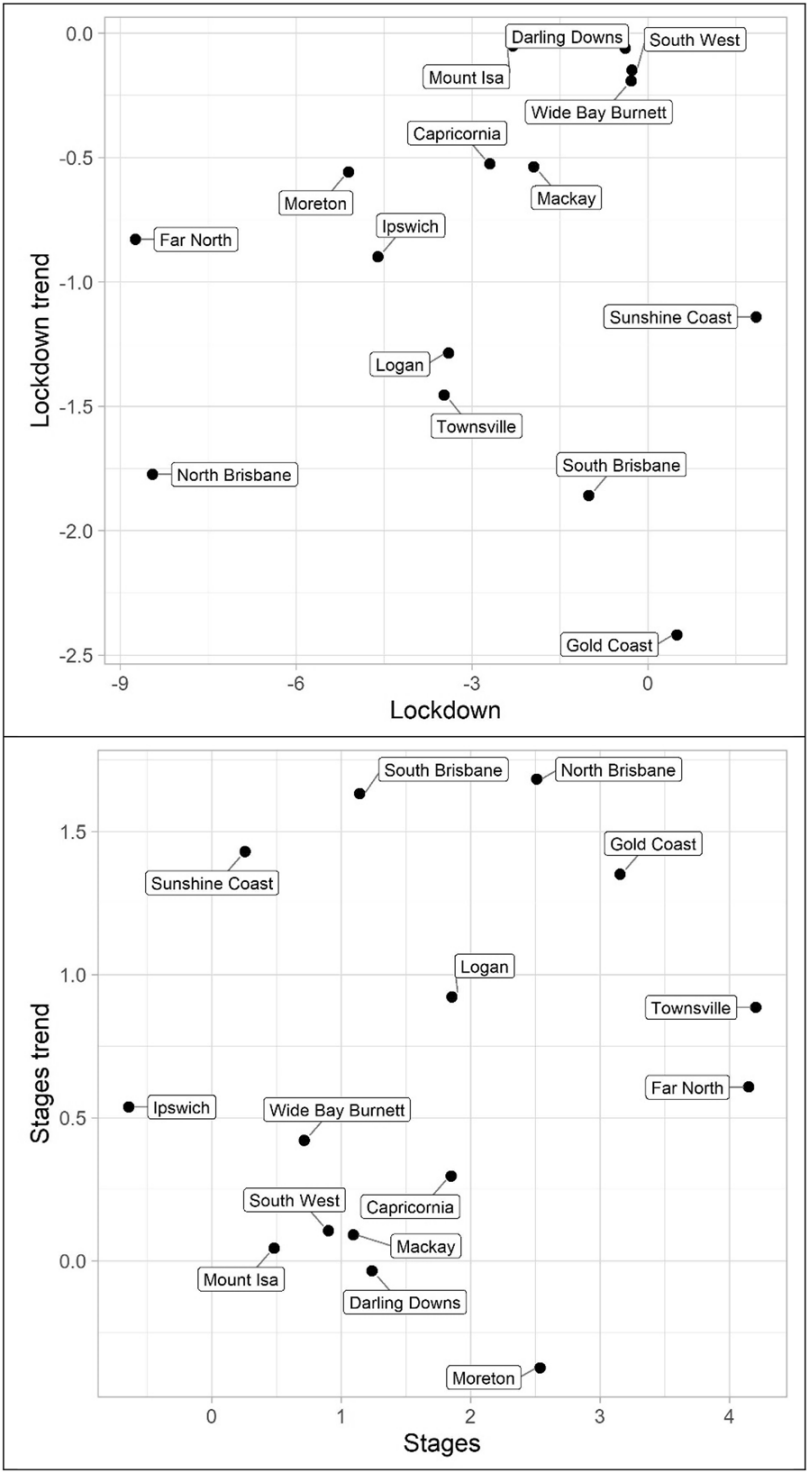
Structural break parameter plots, lockdown and staged relaxation of social restrictions, theft of vehicle

Turning to property offences, Tables 7, 8 and 9 and Figs. 10, 11 and 12 (burglary, theft, and theft of vehicle), theft and theft of vehicle have the expected results for the lockdown immediate and change in trend effects: both negative. In fact, these are the most consistent results found with regards to these expectations and theft have the largest number of statistically significant results. Burglary, however, exhibits an interesting pattern for its lockdown variables. The lockdown trend variables are all negative with almost all of them being statistically significant. The immediate effect of the lockdown, however, has mostly negative parameters but Gold Coast and Sunshine Coast both have statistically significant and large magnitude positive parameters. With regard to the staged relaxation in social restrictions, all property offence variables had a high level of consistency with immediate increases and subsequent increases in trends during this time period. These changes in levels and trends are most consistent with opportunity explanations, compared to social disorder and violent offences. Moreover, this quick return to previous levels of offence is consistent with previous research (Zahnow et al. 2017).

**Fig. 13:**
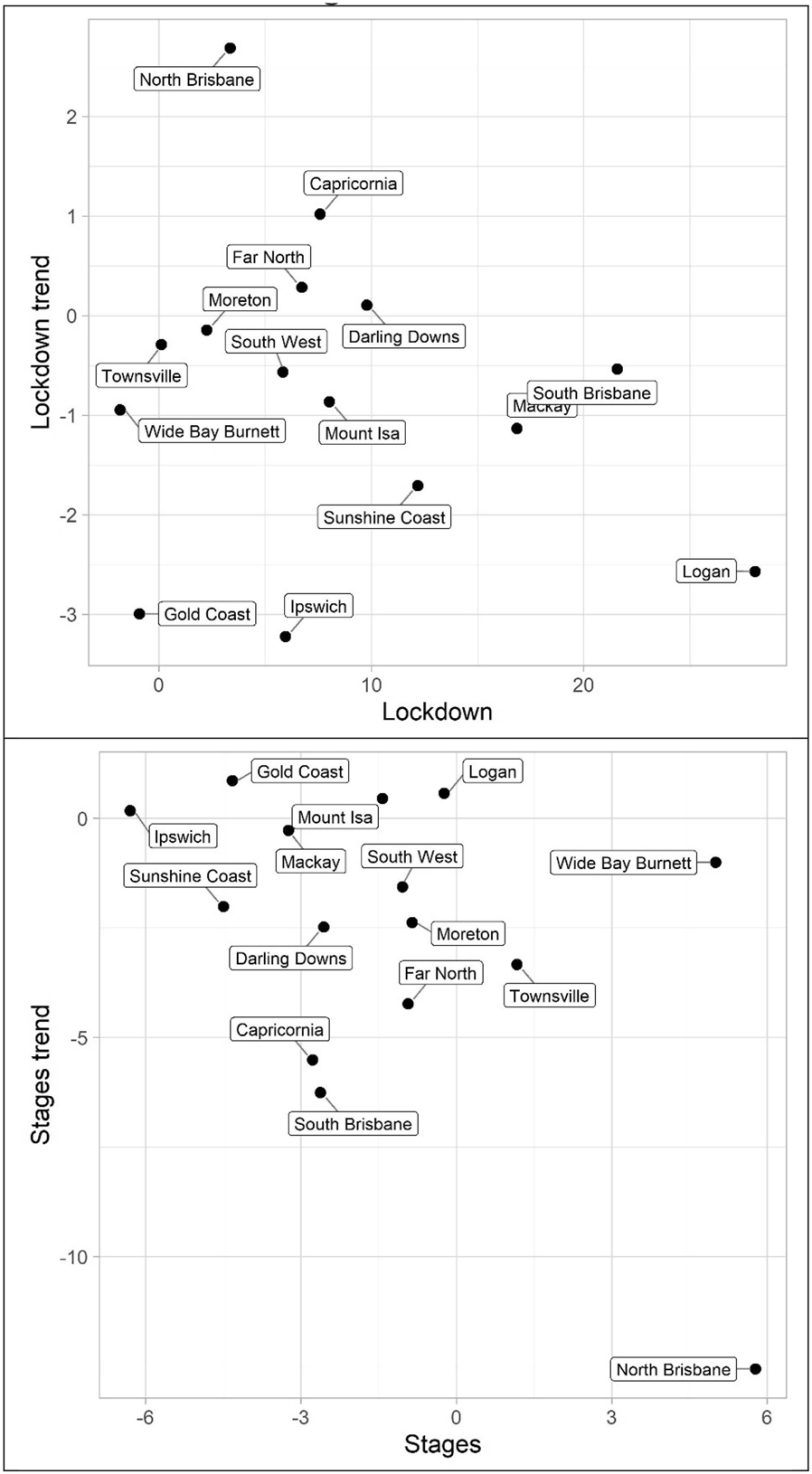
Structural break parameter plots, lockdown and staged relaxation of social restrictions, drugs

**Fig. 14:**
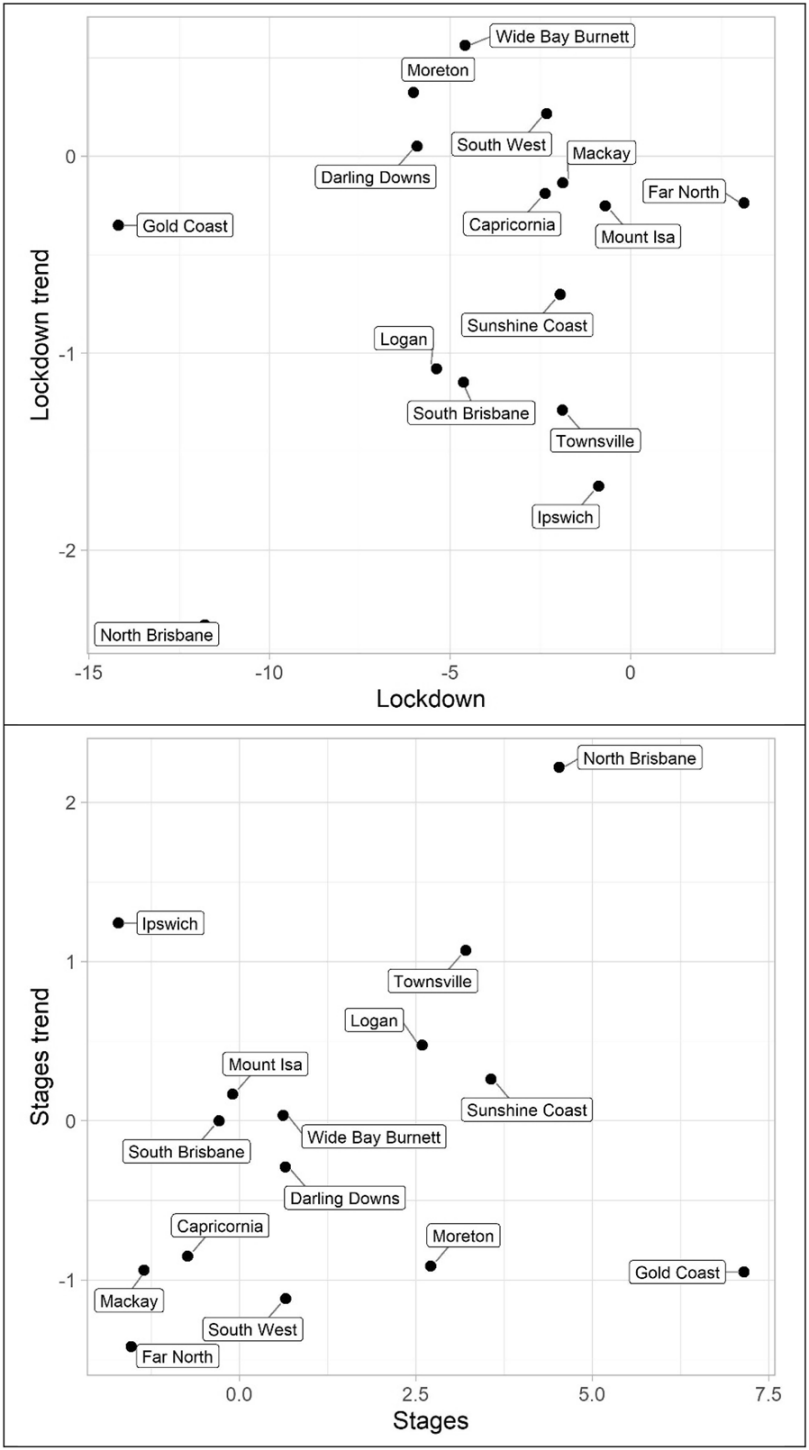
Structural break parameter plots, lockdown and staged relaxation of social restrictions, fraud

**Fig. 15:**
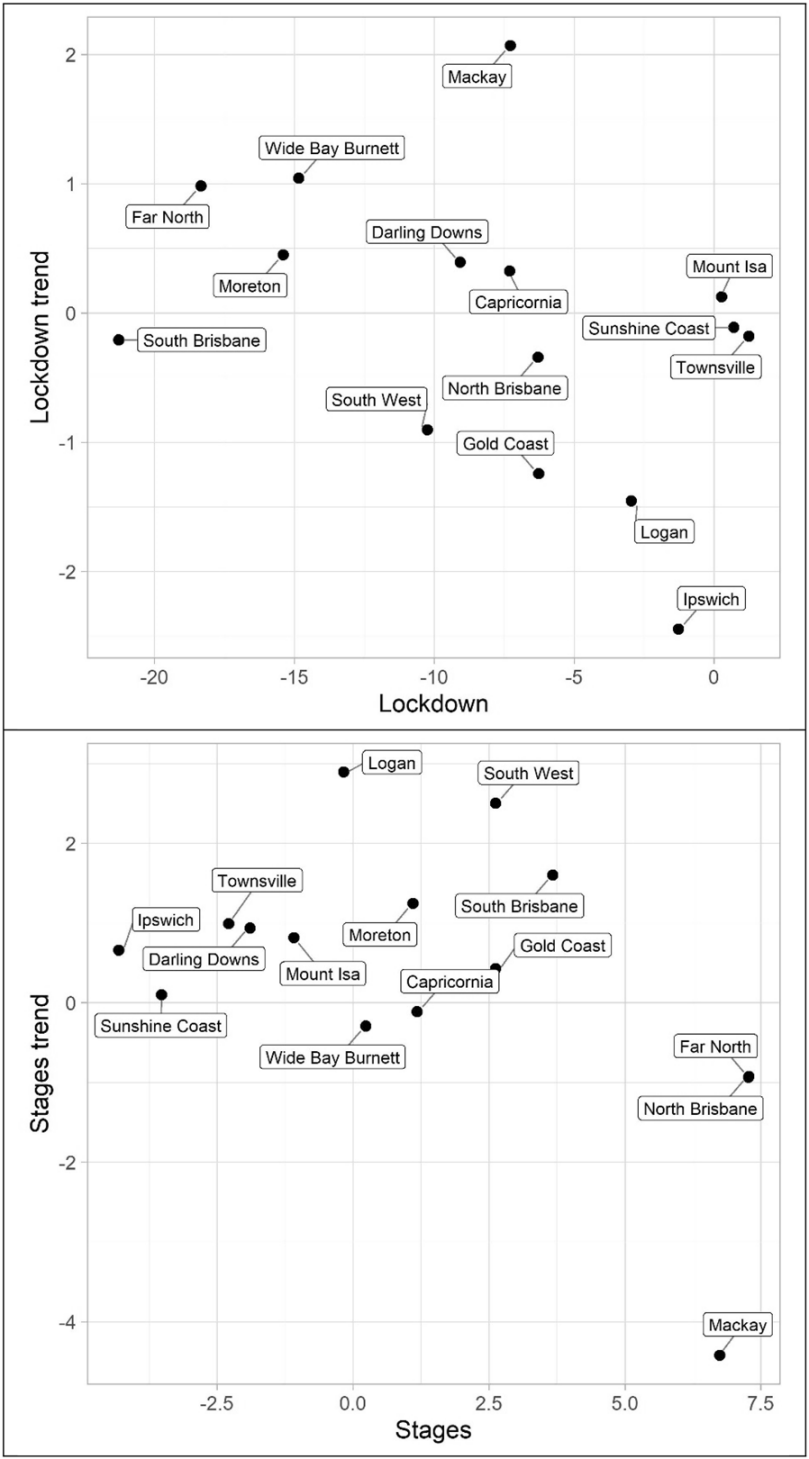
Structural break parameter plots, lockdown and staged relaxation of social restrictions, traffic

Lastly, miscellaneous offences, Tables 10, 11 and 12 and Figs. 13, 14 and 15 (drugs, fraud, and traffic), have varied results. The immediate effect of lockdown on drugs in most QPS districts is positive and statistically significant—all negative parameters are statistically insignificant. Aside from Capricornia and South Brisbane, the trend during lockdown is negative, when statistically significant. Though some districts continued to have positive immediate and trend effects during the staged relaxation of social restrictions (Wide Bay Burnett and South Brisbane, respectively), all other districts exhibited negative parameters for both the immediate effect and subsequent trend. Fraud and traffic, Tables 11 and 12, generally exhibited the expected negative and subsequent positive effects for the lockdown and staged relaxation of social restrictions, respectively, for both immediate changes and changes in trend.

## Discussion

The results of the study offer some interesting areas for further exploration. First, while the findings are relatively consistent with those of other researchers on COVID-19 and crime—crime in general is down during the pandemic—we offer an analysis that considers the post lockdown period. In addition, we use previous years of data for seasonal trend comparisons. As expected, most crime types demonstrate an increase after the easing of restrictions. This is consistent with opportunity theories and predicted by previous research on COVID-19 and crime (Hodgkinson and Andresen 2020). Though it is possible that these results (largely decreases in crime) may be explained by social cohesion/altruism, we do not have any data on changes in social cohesion/altruism to make any inferences here. And given the patterns of change, there is no support for social disorganisation as an explanation for these changes at this time. However, social disorganisation theory tends to be more instructive for longer term explanations of crime patterns (Andresen 2012, 2013; Cantor and Land 1985, 1991).

Second, we conducted this analysis across a large and diverse Australian state. Much of the research on COVID-19 and crime has focused entirely on cities, particularly cities in North America, with more recent international research, as cited above. The analysis of numerous districts that include regional, rural and remote areas, allows us to explore these patterns of crime in different geographical settings and amongst alternative opportunity structures. Indeed, we found different trend patterns in certain contexts.

In the case of the district of Mackay, a large mining community in Northeast Queensland, violence, robbery and assault all increased in concert with the COVID-19 lockdown. These increases are inconsistent with the declines in other districts. Mackay is unique in that it acts as the gateway to the Bowen Basin, which is 60,000 km^2^ of coal reserves—the largest mining area in Australia. Mackay is also home to a large number of ‘donga’ communities which are pop-up residences for mining workers and largely house men only. During the lockdown, Mackay suffered a huge loss of mining jobs due to the drop in the cost of coal internationally and the COVID-19 related restrictions put in place on mining workers (Szabo 2020; Whiting 2020). This situation differs greatly from other Queensland districts. While we can only offer conjecture at this point, we believe that the loss in jobs may have left a predominantly male workforce out of work and in the presence of a lot of other men who are also out of work. This could increase opportunities for assault or alcohol/drug-related violence (Carrington et al. 2012). Indeed, Mackay did experience one of the most significant increases in drug-related offenses during the lockdown. In addition, this could create additional opportunities for domestic violence for men who are out of work and at home for extended periods of time with their victims (United Nations 2020); however, this depends on where the families of these workers live given the fly-in/fly-out nature of the mining communities. Further research on the impact of COVID-19 in this area, and other non-urban or natural resource dependent communities is necessary.

Third, we found that depending on the nature of ownership and use of space in cities, some cities may be at an increased risk for certain types of crime. The Sunshine Coast and Gold Coast, for example, experienced sharp initial increases in burglary at the beginning of the lockdown, followed by the expected decreases. A possible explanation for the different lockdown effects for burglary in the Gold Coast and Sunshine Coast, may be that these two areas are close to the primary urban area of Queensland (Brisbane) and contain a lot of vacation homes and vacation rentals. Unable to travel to these locations during the lockdown (initial lockdown restrictions limited travel to under 50 km), these homes may have been suitable targets in that they had no guardianship. However, once these targets had been burglarized, they were no longer suitable as owners and renters were unable to travel to these homes to replace what had been stolen. Until owners, security, and police could respond to these increased opportunities for burglary, which would be delayed by the lack of presence and ownership in these tourist spaces (Mawby 2015) this increase in burglaries is not surprising. In fact, this is precisely what occurred in Vancouver, Canada with regard to commercial burglary until security was increased and police increased activities in these areas (Hodgkinson and Andresen 2020)—this is generally consistent with changing opportunity structures and crime (Hodgkinson et al. 2016; Hodgkinson and Andresen 2019). Again, we speculate this is the case and further research into the impact of crime on tourist locations may be necessary.

Fourth, we did not find the expected shift in border related traffic offenses that we would have expected during and after the social restrictions. The districts that bordered other states (Mount Isa, South West, Darling Downs, Ipswich, Logan, and Gold Coast) did not experience an increase in traffic-related offences because of inter-state travel restrictions. This may simply be a result of the great distances between states in Australia and the increased effort necessary to break these laws considering the presence of police along these borders and that all states in Australia imposed strict domestic travel restrictions during this time. It may also be the case that changes in police operational practices during COVID (Crockford and Lynch 2020; Queensland Police Service, 2020) have led to decreased reported incidents. It may be interesting to see if other smaller countries that put in place border control, witnessed any shift in traffic-related offenses.

Fifth and finally, the study demonstrates an increase in drug-related offenses post-lockdown in 13 of the 15 districts. 9 of these increases were significant. Mt Isa, Logan and Mackay experienced that greatest magnitude increases in these offenses in comparison to the baseline in the early stages of lockdown. While Logan and Mt Isa decreased in drug-related offenses after the relaxation of restrictions, Mackay remained steady. As mentioned above, this may be a result of the loss of mining jobs in the area due to the drop in coal-prices internationally. The overall increase in drug use at the point of lockdown across most of the districts, could indicate an increase in self-medicating behaviours to the insecurity of the pandemic. While the social restrictions have had the expected impact on the accessibility of drugs on the global market, as many routes for drug trafficking have been interrupted, this has not prevented people from stockpiling and using less pure forms or lesser alternatives such as cannabis (which is still illegal in Australia) to self-medicate (UNODC 2020). The increase in drug use across Queensland during the pandemic is probably the most contradictory finding to opportunity theories, as it demonstrates that, increases in effort or decreases in opportunities, do not dissuade those who are dealing with self-medicating behaviour or addiction.

As with all research, ours is not without limitations. First and foremost, our analyses rely on offences reported to the police. Though the lack of reporting to the police is long-standing and well-known (Bulwer 1836; Perreault 2015), reporting of offences to the police is higher in Australia than many western countries: 54% (assault), 39% (sexual assault), 90% (theft of vehicle), 75% (residential burglary) (Australian Bureau of Statistics 2018). However, there is no way to know if reporting rates have remained constant during the pandemic; for example, police and other social services have modified practices because of social distancing requirements and some victims may be less likely to report criminal occurrences to avoid contact, more generally. There is little that can be done regarding this limitation but given the higher level of reporting in Australia it is of a lesser concern than other countries. Second, though it would not be feasible across an area as large as Queensland, Australia, we do not consider local area variations for the impact of COVID-19 on crime. As with previous research, cited above, there may be variations within districts that may prove to be important. However, we believe this large-scale analysis sets the stage for further investigation into some of the identified areas such as communities reliant on natural resources, and tourist destinations. Third, through domestic violence has been reported to be on the rise in Queensland, Australia (Bavas 2020), and important in the context of pandemics (Mohler et al. 2020; Parkinson 2019; United Nations 2020), we are unable to disentangle the violence data in this study as domestic violence incidents are rarely presented separately in open data sources. And fourth, though we have been able to show regional variations, our regions (police districts) are still quite large. Smaller areas of analysis should be undertaken to see if the effects of COVID-19 vary by neighbourhood.

In addition to addressing these limitations, future research should move beyond investigations of the impact of COVID-19 on crime in urban centres. Significant populations, upwards of 20 to 30 percent, continue to live in rural areas, even in developed countries such as Australia, Canada, the United States, France, Germany, and Japan (Statistics Canada, 2012). In addition, in countries like Australia and Canada, crime rates are often higher in rural and regional areas (Hogg and Carrington 2006; Ruddell 2016). In the context of COVID-19, these areas suffer greater harms because of decreased access to social/medical services. Moreover, opportunity approaches can only go so far in understanding the (changing) patterns of crime, particularly with regard to exceptional events. Longer term effects need to be tested in order to properly assess the opposing predictions of social cohesion/altruism and social disorganisation theory.

## Conclusion

We explored the rates of different crime types across the state of Queensland, Australia before, during and after the COVID-19-related lockdown. We find, as predicted by opportunities theories, that most crime types, except for drug-related offenses, decreased during the lockdown and subsequently increased after the restrictions were lifted. However, in particular contexts, certain crime types increased as a result of the lockdown. These shifts appear to be affected by loss of jobs and self-medicating behaviours, as well as the number of suitable targets in tourist destinations. We suggest that further research examine non-urban or unique contexts to explore these findings greater detail. However, we believe these findings are useful in understanding the impact of a global pandemic on crime trends.

## Data Availability

All data are open source and available from the authors upon request.

## Footnotes

1 These offences include public nuisance, disorderly/offensive behaviour, public urination, and so on.

2 Unfortunately, no information regarding what is included in other violence is available.
